# Effects of intercropping with legume forage on the rhizosphere microbial community structure of tea plants

**DOI:** 10.3389/fmicb.2024.1474941

**Published:** 2024-11-25

**Authors:** Yuhang Jiang, Xiaoqin Lin, Wenxiong Lin

**Affiliations:** ^1^College of Life Science, Longyan University, Longyan, China; ^2^Fujian Provincial Key Laboratory of Agroecological Processing and Safety Monitoring, Fujian Agriculture and Forestry University, College of Life Sciences, Fuzhou, China; ^3^Key Laboratory of Crop Ecology and Molecular Physiology (Fujian Agriculture and Forestry University), Fujian Province University, College of Life Sciences, Fuzhou, China; ^4^School of Resource Engineering, Longyan University, Longyan, China

**Keywords:** tea plant, intercropping, forage legume, rhizosphere effect, microbial community structure

## Abstract

**Context:**

Intercropping in agriculture is crucial for addressing challenges in intensive tea farming. Forage legumes reduce fertilizer dependence and significantly boost productivity. Currently, intercropping with legumes enhances the environmental conditions of tea plantations and improves tea quality.

**Objective:**

However, the comprehension of the rhizosphere’s impact on the associated microbes and the community structure of tea plants is still somewhat constrained.

**Methods:**

Hence, four distinct planting methodologies were examined: Monoculture cultivation of Tieguanyin tea plants (MT), Laredo forage soybean (*Glycine max* Linn.) without partitioning in conjunction with tea (IT), intercropping with tea using plastic partitions (PPIT), and intercropping with tea facilitated by net partitions (NPIT). An absolute quantitative analysis of soil phospholipid fatty acids, labeled with the rhizosphere microbial characteristics of tea plants, was conducted through multi-ion reaction monitoring (MRM). The bacterial and fungal communities were anticipated utilizing the FAPROTAX and FUNG databases, respectively. Gas chromatography was employed to ascertain greenhouse gas emissions across diverse root interaction cultivation systems.

**Results and conclusion:**

The rhizospheric influence culminated in a 44.6% increase in total phospholipid fatty acids (PLFAs) and a remarkable 100.9% escalation in the ratio of unsaturated to saturated fatty acids. This rhizospheric enhancement has significantly potentiated the ecological functionalities within the bacterial community, including xylanolysis, ureolysis, nitrogen respiration, nitrogen fixation, nitrite respiration, nitrite ammonification, and nitrate reduction. Mycorrhizomonas, encompassing both ectomycorrhizal and arbuscular forms, has notably colonized the rhizosphere. The interspecific mutualistic interactions within the rhizosphere have resulted in a significant enhancement of plant growth-promoting bacteria, including *allorhizobium*, *bradyrhizobium*, *rhizobium*, *burkholderia*, *gluconacetobacter*, and *gluconobacter*, while concurrently reducing the prevalence of pathogenic microorganisms such as *xanthomonas*, *ralstonia*, *fusarium*, and opportunistic fungi responsible for white and soft rot. The intercropping system showed lower total greenhouse gas emissions than monocultured tea plants, particularly reducing soil CO_2_ emissions due to complex interspecific rhizosphere interactions. This tea/legume intercropping approach promotes a sustainable ecosystem, enhancing microbial biomass and vitality, which helps suppress rhizospheric pathogens.

**Significance:**

These findings are instrumental in enhancing our comprehension of the pivotal practical implications of rhizosphere intercropping, thereby optimizing the structure of rhizosphere communities and alleviating the impact of greenhouse gases within croplands.

## 1 Introduction

As one of the three most popular nonalcoholic beverages in the world, tea has important economic, health, and cultural value (Xia et al., 2017). China was the first country to cultivate tea [*Camellia sinensis* (L.) O. Ktze.] and is the world’s largest tea-producing region. At present, China accounts for more than 40% of the world’s tea production, with an area of 3.37 × 10^6^ ha (Food and Agriculture Organization, 2020). Large-scale intensive monoculture farming dominates most tea plantations. Simplifying continuous monoculture systems and overusing agrochemicals and pesticides accelerated soil degradation and increased the risk of pest outbreaks and pollution (Tsioumani, 2019; Rockström et al., 2017). Currently, a number of continuous cropping obstacles caused by continuous tea monoculture, such as soil degradation, pest outbreaks, and decreases in tea yield and quality, have seriously challenged the sustainable and healthy development of China’s tea industry (Li et al., 2017; Arafat et al., 2017).

Intercropping technology has been widely used because of its many advantages, including improving and stabilizing crop yield (Li et al., 2021), efficiently using resources (Mao et al., 2015), inhibiting pests and diseases (Boudreau, 2013), alleviating climate change (Glaze-Corcoran et al., 2020), and increasing biodiversity on farmlands (Yang et al., 2021). Based on the spatial characteristics of the system community, it is considered a three-dimensional form of farming that is efficient in using space and resources and is becoming increasingly important in modern agricultural production (Yang et al., 2021). Intercropping tea and green manure is considered to be a habitat management practice or an environmental enhancement technology for improving soil fertility, conserving soil and water, and maintaining soil health (Shi et al., 2022; Wang et al., 2022). As a high-value resource with high efficiency in the combination of planting and cultivation of land and intercropping, forage legumes have great advantages in terms of weight reduction and efficiency, soil and water conservation and resource efficiency (Espinoza et al., 2020). Currently, forage legumes are widely used as functional crops in tea plantations in tea-fertilizer intercropping systems (Peng et al., 2008; Feng and Sunderland, 2023).

Microbial communities in the rhizosphere are essential for the health and productivity of plants, and microbial aggregation and dissipation are controlled by biotic and abiotic factors (Philippot et al., 2024). Many studies have shown that the soil environment has a profound effect on the aggregation of rhizosphere bacterial and mycorrhizal fungal communities (Philippot et al., 2013). It is well known that the lack of fertility of the rhizosphere is the major cause of the decline in tea yield and quality. Pests and diseases are uncontrollable factors that directly threaten the health of tea plants, and these uncontrollable factors are more likely to occur in a single cultivated tea garden (Hazarika et al., 2009). Reasonable intercropping in tea gardens is conducive to improving the biodiversity level of the tea garden system and reducing the risk of disease outbreaks by improving the ability of the tea plant to resist pests and diseases (Dai, 2020). Research on pest and disease prevention and control in intercropped tea gardens has been conducted in recent years and has focused more on influencing aboveground interactions. However, little attention has been given to the feedback response of the rhizosphere effect of underground intercropping on the resistance to soil diseases during the process of tea cultivation to resist soil diseases from infringement (Fu et al., 2016). Related studies suggest that an important factor in determining the structure and disease-suppressing function of the microbial flora of the rhizosphere is the nature of the root exudates. Intercropping can be considered an effective strategy for regulating the components of root exudates and improving the overall disease inhibitory capacity of the soil (Gu et al., 2020). The rhizosphere effect of tea/legume intercropping has a significant impact on the number and type of metabolites that are secreted in the rhizosphere of tea plants (Huang et al., 2019). Therefore, the rhizosphere effect of the intercropping process is highly important for influencing the microbial community structure of the tea rhizosphere and regulating soil diseases to better understand the ecological value of intercropping.

Global climate change is influenced by soil fertility and carbon sequestration. The preservation of soil fertility and the reduction in carbon emissions from agricultural production are highly important for mitigating global warming (Yang et al., 2021). Soil is a major source of greenhouse gases, and the emission of carbon dioxide from soil is a process driven by microbes. Soil carbon accumulates primarily through the decomposition of plant litter, and soil microbes are responsible for the majority of carbon processing in plants (Gougoulias et al., 2014). Therefore, the formation and decomposition of sequestered soil carbon are greatly enhanced by soil microbes, which has a significant impact on carbon dioxide emissions (Ren et al., 2022). The fixation of soil organic carbon in an intercropping system is highly important for reducing greenhouse gas emissions (Cong et al., 2015). Soil phospholipid fatty acid is an important component of the living microbial cell membrane and an important biomarker for the study of changes in the structure of the microbial community (Kujur and Patel, 2014). Therefore, this study quantified phospholipid fatty acids, the main characteristic markers of rhizosphere soil, and explored the effect of rhizosphere intercropping on microbial community structure at the overall level. The bacterial 16S rRNA gene sequence and fungal ITS gene sequence were sequenced by second-generation high-throughput technology to understand the diversity and characteristics of rhizosphere microorganisms in the intercropping rhizosphere. Simultaneously, the microbial functions of the bacterial and fungal communities were predicted and analyzed to study the effect of the rhizosphere on the main ecological functional flora. Multiple comparative analyses revealed the response patterns of plant growth-promoting bacteria and pathogenic bacteria under the regulation of the rhizosphere effect. Finally, the importance of the rhizosphere effect in reducing carbon emissions was clarified by the difference in greenhouse gas changes between different cropping systems.

## 2 Materials and methods

### 2.1 Field test conditions

**Figure 1:**
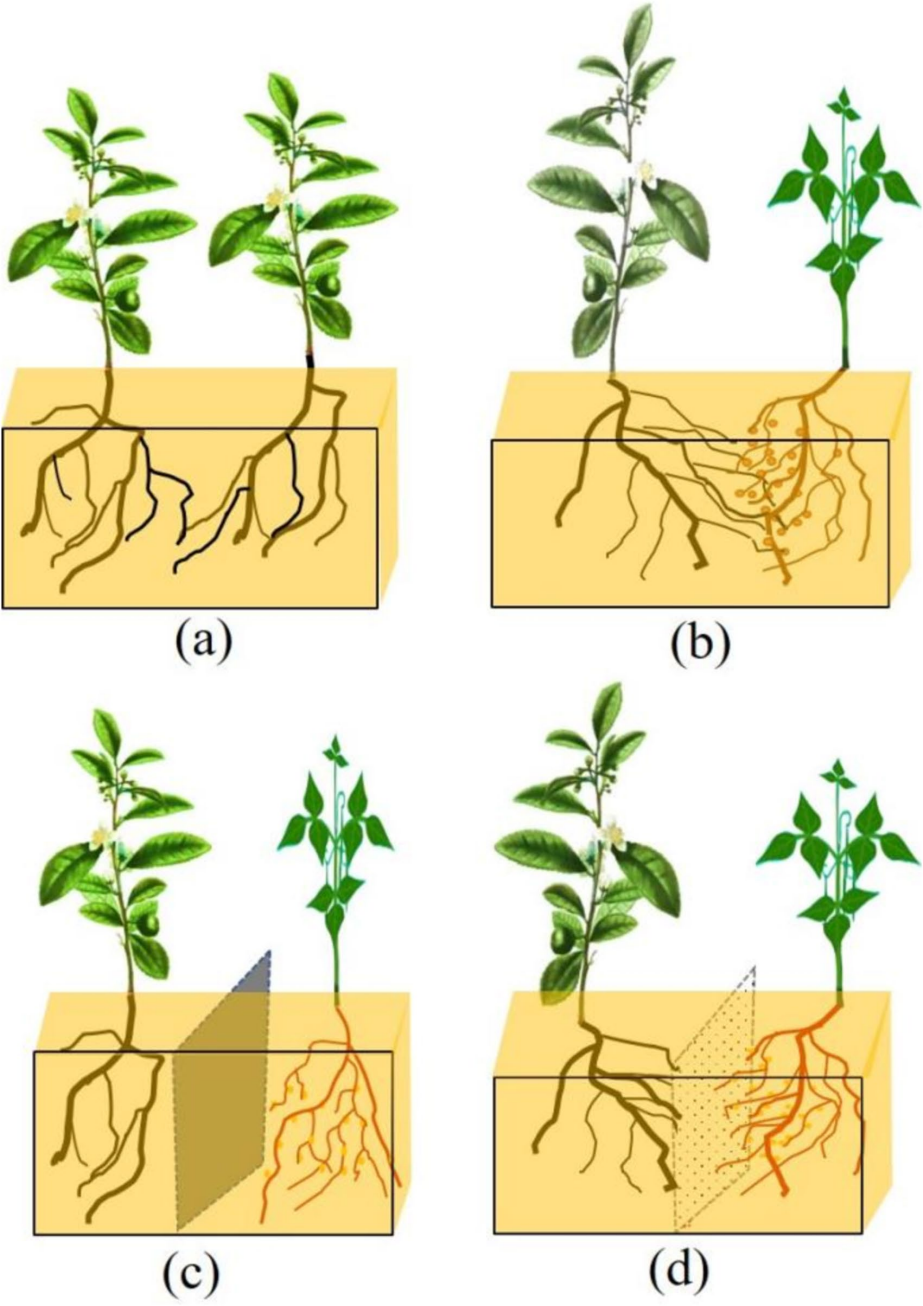
Test field experiment for different cultivation between tea plant and forage legume. **(A)** MT, Monocropping tea plant. **(B)** IT, Non-partition in intercropping tea plant. **(C)** PPIT, Plastic partition in intercropped tea plant. **(D)** NPIT, Netted partition in intercropped tea plant.

In this study, two-year-old Tieguanyin [*Camellia sinensis* (L.) O. Ktze.] was selected as the test material, which was derived from the tea seedling cultivation base (24°57’N, 117°4′E) in Yaxing Village, Anxi County, Fujian Province. The forage legume used was Laredo soybean (Jiang et al., 2019). The field experiment was carried out at the seedling base in October 2019. The test field (length 250 cm, width 150 cm, depth 50 cm) was divided into four treatments: MT (monocropping tea plant), IT (no-barrier intercropping), PPIT (plastic partition in intercropping), and NPIT (netted partition in intercropping). PPIT was separated by ethylene vinyl acetate copolymer (EVA) plastic, which has excellent sealing performance and corrosion resistance. The net partition material used was a 300 mesh nylon net in the NPIT. The cultivation conditions of the experimental plots are shown in Figure 1. Water and fertilizer management in the field was carried out according to the local tea nursery cultivation method, and pests and diseases were prevented regularly during the intercropping growth period. Rhizosphere soils were collected from four groups of tea rhizosphere soils (MT, IT, PPIT, and NPIT) subjected to different cultivation treatments in Yaxing village, Anxi County, in March 2022. The residual roots, litter and particulate matter in the samples were removed, and the quartering method was used for random sampling for subsequent sample detection.

### 2.2 Determination of phospholipid fatty acids in tea rhizosphere soil

Extraction of soil phospholipid fatty acid lipids was performed by alkaline esterification (Wu et al., 2009). Subsequent analyses were performed using GC–MS multiple reaction monitoring (MRM) for analyzing the PLFAs in the soil. First, 33 standard mixtures of FAMEs (Dr. Ehrenstorfer, Germany) were used to optimize the gas chromatography conditions. The solution was diluted with n-hexane to 5 μg/mL for full-scan Q3 analysis. The chromatographic conditions were set up as follows: The determination was performed by gas chromatography–mass spectrometry (GC–MS TQ8040, Shimadzu, Japan). Splitless injection was used with 99.999% He as the carrier gas passing through the column (SH-Rxi-17Sil MS, Shimadzu, Japan) at 1.4 mL/min. The sample collection time was 0.5 min, and the flow rate was controlled at 42.8 cm/s. The temperature program used to heat the column box was as follows: 40°C was increased to 200°C at a rate of 10°C/min and then increased to 310°C at a rate of 6°C/min for 2 min. An ionization energy of 70 *eV* was used for electron bombardment in the mass spectrometer. The temperature of the inlet and the interface was 280°C, and the temperature of the ion source was 200°C. The solvent removal time was 1.50 min, and the mass spectrometry scan range was from 45 to 500 m/z. The conditions of the TIC (Supplementary Figure S1) met the requirements for subsequent quantitative analysis by MRM. Q3 data derived from the standard solution were used for postrun analysis data analysis. The precursor ions of each of the fatty acid methyl esters were determined from the scan data file. The products of each group of fatty acid methyl esters were generated. The range of the collision voltage and the voltage interval were set by the MRM Optimization Tool Optimization Tool, and the product ion scanning method and batch table were generated with different collision voltages. The collision voltage was automatically optimized to obtain the optimal collision voltage and the optimal ion abundance ratio of each component (Supplementary Table S1). The optimization component information has been registered in the Smart Database table, and finally, an MRM analysis method of the fatty acid methyl ester component was created. A gradient fatty acid methyl ester standard mixture of 0.05, 0.10, 0.20, 0.50, 1.00, and 5.00 μg/mL was determined by the newly established MRM method, and the corrected standard curve equation was obtained (Supplementary Table S2).

### 2.3 Rhizosphere microbial diversity analysis

Total DNA was extracted from the samples using a kit (Omega Bio-Tek, Norcross, GA, United States). Specific primers for the conserved regions of bacterial 16S rDNA v3-v4 and fungal ITS (bacterial primer pairs: 338F and 806R; fungal primers ITS1 and ITS4) were amplified by PCR. The product was purified, quantified and homogenized for library construction. Double-ended sequencing was performed on an Illumina NovaSeq 6,000 instrument. The raw reads were filtered using Trimmomatic v0.33 software. Clean reads were obtained using Cutadapt 1.9.1 software to identify and remove primer sequences. The clean reads of each sample were overlapped using Usearch v10 software. Chimeras were identified and removed using UCHIME v4.2 to obtain effective reads. According to different levels of similarity, all the sequences were clustered according to OTUs (classification operation units) to obtain effective OTUs.

### 2.4 Predictive analysis of microbial function

Using SILVA as a reference database, the OTUs were annotated by the naive Bayes classifier, and the corresponding species classification information was obtained. Then, the community composition at different taxonomic levels, including phylum, class, order, family, genus and species, was determined. QIIME software was used to generate species abundance tables at different classification levels. The FAPROTAX (functional annotation of transgenic taxa) database was used to perform rapid functional screening and grouping of 16S bacterial data from terrestrial ecosystems (Sansupa et al., 2021). Based on the OTU classification table of bacterial 16S, the FAPROTAX database was used to predict the biogeochemical cycling process of soil samples for functional annotation (Louca et al., 2016). Based on the OTU annotation classification table of fungal ITSs, the FUNGuild (fungi functional guild) database was used to group the functions of fungal community structure in the absorption and utilization of environmental resources (Nguyen et al., 2016). Thus, the nutritional function grouping table of the mushrooms was obtained.

### 2.5 Determination of greenhouse gas emissions in the intercropping system

#### 2.5.1 Collection of gas samples

**Figure 2:**
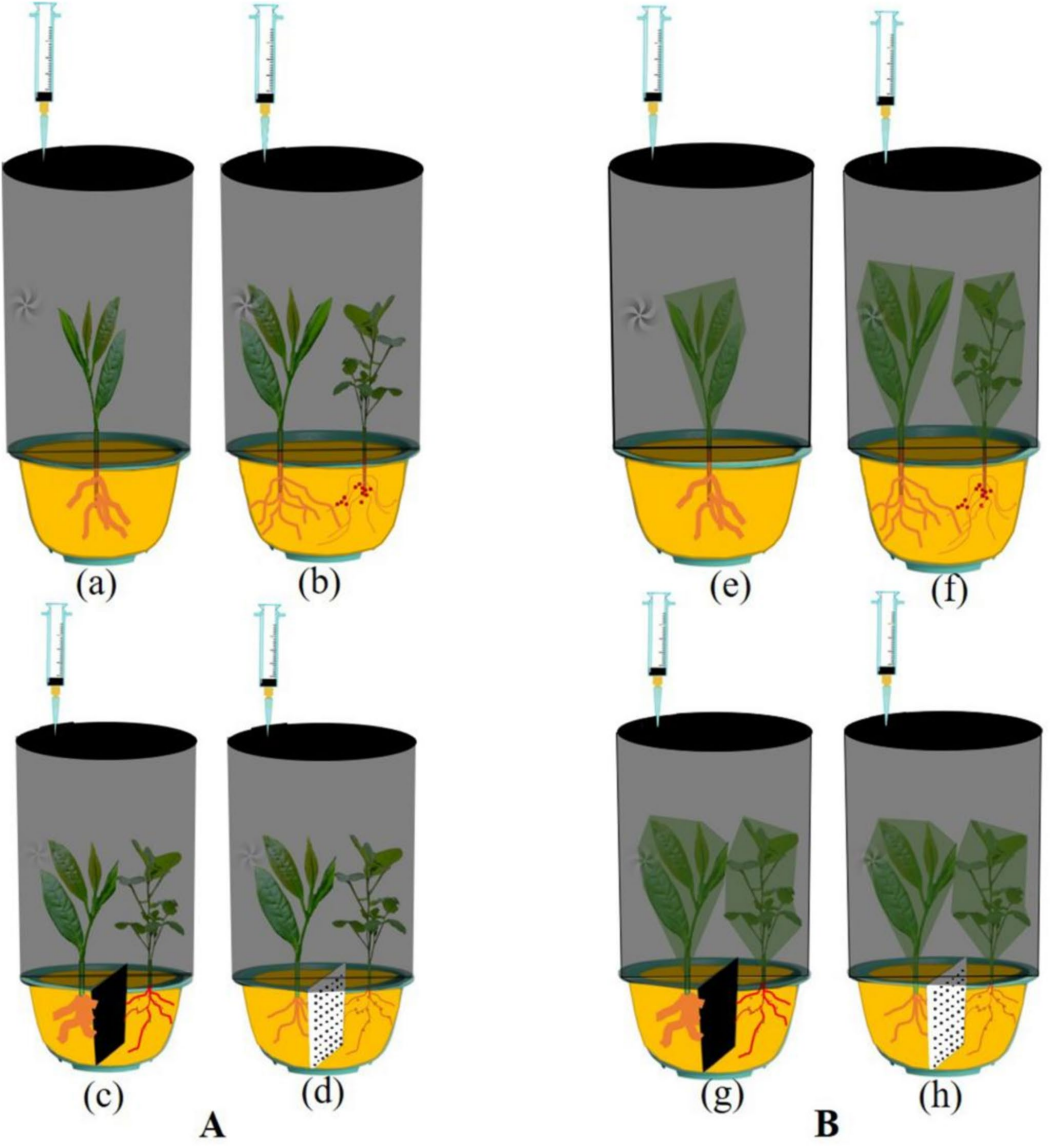
Catchments of greenhouse gas in tea/forage legume intercropping. **(A)** Greenhouse gas collection in different cultivation systems, including MT, IT, PPIT, and NPIT. **(B)** Greenhouse gas collection from soil part in different cultivation systems, including MT, IT, PPIT, and NPIT.

A homemade device (with an inner diameter of 28.5 cm and inner height of 48 cm) with an external 150 mL injection syringe with a built-in air blower was used for the collection of gas samples (Figure 2). One tea plant and one forage legume were cultivated in each pot, and the plant spacing was kept consistent. GHG sampling was conducted using four growing system treatments. Two-year-old tea plants and 90-day-old forage legumes were grown under the same conditions. Dark conditions are used throughout the collection process on June 28, 2022, from 9 to 11 a.m. Gas samples were collected from the same set of treated chambers every 10 min for a total of 3 times. Gas was collected from each cultivation system for a total of 30 min. Before each sampling, the sample was pulled up and down a few more times to ensure uniformity of the gas sample. The cap was quickly attached to the injection syringe nozzle of 150 mL of extracted gas. In addition, the gasket film is wound at the seam to prevent gas leakage. Then, the GHGs of both soil and aboveground parts of MT, IT, NPIT, and PPIT were collected, respectively (Figure 2Aa–d). At the same time, the above-ground the plant parts of four cultivation modes were bagged, and then used for greenhouse gas collection (Figure 2Bd–h) for determine the amount of GHGs emitted from the soil in the growing system. Each treatment had three biological replicates.

Greenhouse gas chromatographic conditions: A gas chromatograph (SCION 456i) was used to detect greenhouse gases. The column oven was equipped with four special chromatographic columns (HayesepD, 2.0 m × 1/8″ × 2 mm Inert, 80/100 mesh; Porapak QS, 2.0 m × 1/8″ × 2 mm Inert, 80/100 mesh; HayesepN, 0.5 m1/8″ × 2 mm Inert, 80/100 mesh; HayesepN, 0.5 m1/8″ × 2 mm Inert, 80/100 mesh) for the analysis of greenhouse gases. The temperatures of the front packed column inlet and column oven were 150 and 50°C, respectively. The column pneumatic device is equipped with a front-mounted electronic flow controller (flow: 30 mL/min, pressure: 6.00 Psi), medium electronic flow controller (30 mL/min, 20.00 Psi) and rear flash port electronic flow control (identical to the front electronic flow controller). The monitor is equipped with a prehydrogen flame ionization monitor and an intermediate electron capture monitor at 250 and 300°C. The flow rates were 10, 30, and 300 mL/min for the N_2_ tail gas, H_2_ combustion gas, and air, respectively. The flow rate of the electron capture detector tail gas N_2_ was 30 mL/min. The flow pressures of 99.999% N_2_ blast gas, 99.999% H_2_ combustion gas and combustion air (21% O_2_, 79% N_2_) are 0.70, 0.50, and 0.45 MPa, respectively. A total of 3.5 min was required for sample determination. The injection volume of the quantitative valve was 5 mL.

Measurement of GHG Emissions: The appropriate dilution multiples were set in the instrument software according to the chromatographic conditions, and the standard curves of the greenhouse gas standards CH_4_ (2.80 ~ 14.80 μg/mL), CO_2_ (408.00 ~ 2007.00 μg/mL), and N_2_O (0.33 ~ 1.25 μg/mL) were plotted in sequence. The instrument software corrected the three standard curves to produce the following standard working curves: Y_CH4_ = 31.928x + 5.20073, R^2^ = 0.9985; Y_CO2_ = 30.679x + 1407.7, R^2^ = 0.9998; and Y_N2O_ = 3216.8x + 1238.8, R^2^ = 0.9999. The samples were then sequentially injected into the injection port to obtain the corresponding greenhouse gas concentration (μg/mL).

### 2.6 Data analysis

One-way analysis of variance (one-way ANOVA) was performed on the values between groups using SPSS 18.0 software (SPSS Inc., United States). Independent samples t tests were used to compare the values between the two groups. The level of significance of the difference was *p* < 0.05. Origin Pro 2019b (Origin Lab, United States) software was used to construct nutrient functional fungal community radar plots. The least significant difference (LSD) method was used to perform multiple comparison analysis of variance on the rhizosphere growth-promoting microbes and pathogenic microbial community, and the significance level was *p* < 0.05. The Micro Bioinformatics platform^1^ was used for cluster analysis.

## 3 Results

### 3.1 Changes in PLFA characteristic marker microbes in the intercropping rhizosphere

**Figure 3:**
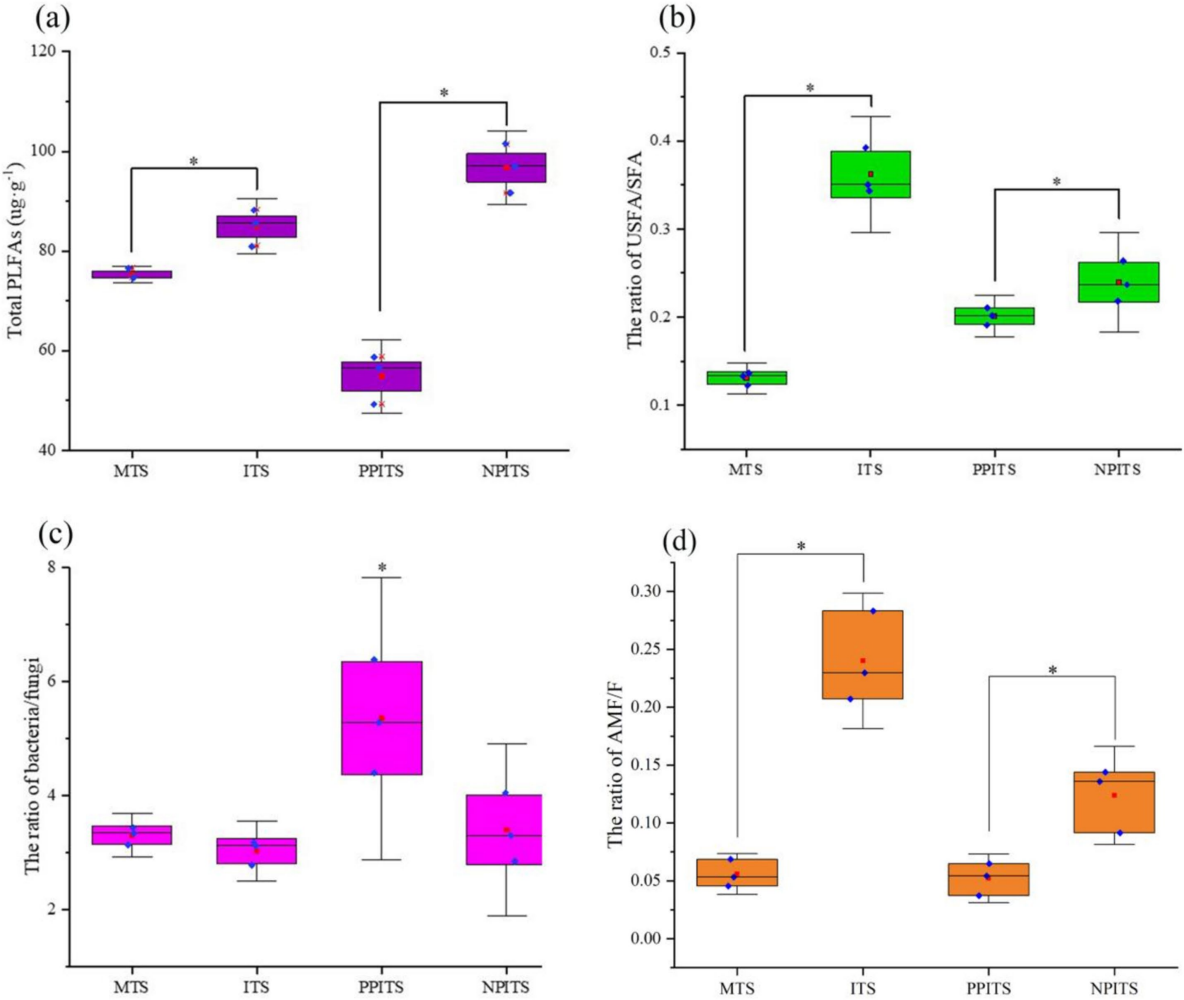
Microbial distribution under phospholipid fatty acid labeling in soil during rhizosphere intercropping. **(A)** Total PLFAs in different tea rhizosphere soil. **(B)** The ratio of unsaturated fatty acid/saturated fatty acid in different tea rhizosphere soil. **(C)** The ratio of bacteria/fungi in different tea rhizosphere soil. **(D)** The ratio of arbuscular mycorrhizal fungi/fungi in different tea rhizosphere. The red square indicated the Mean-Value.

Thirty-one fatty acids quantified by MRM were characterized (Table 1). The labeled species mainly included bacteria, fungi, aerobic gram-negative bacteria, arbuscular mycorrhizal fungi, saprophytic fungi, actinomycetes, gram-positive bacteria, yeasts and protozoa. Compared with those in the MTSs, the percentage of fatty acid-labeled bacteria in the ITSs increased by 8.9%, the percentage of fungi increased by 0.3%, and the percentage of arbuscular mycorrhizal fungi increased significantly by 329.5% (*p* < 0.01). Compared with those in PPITS, the percentage of fatty acids labeled with bacteria in NPITS significantly increased by 49.7% (*p* < 0.01), the percentage of fungi increased by 11.6% (*p* < 0.01), and the percentage of AMF significantly increased by 100.8%. From the perspective of the total fatty acid content (Figure 3), the total fatty acid content in the ITSs was 11.1% greater than that in the MTSs (*p* < 0.05). Overall, 78.1% (*p* < 0.001) more total fatty acids were detected in NPITS than in PPITS, while the total fatty acids in PPITS decreased significantly compared with those in MTS. The ratio of unsaturated fatty acids to saturated fatty acids in the ITS group was 177.2% greater (*p* < 0.001) than that in the MTS group. There was also a 24.5% increase in the NPITS ratio (*p* < 0.05). Compared with that in the MTSs, the ratio of bacteria to fungi in the ITSs did not change significantly, while compared with that in the PPITSs, the NPITS decreased significantly, by 36.6% (*p* < 0.05). Moreover, the proportion of AMF in the intercropping system increased significantly, by 330.1% (*p* < 0.01).

**Table 1:** Effects of different cultivations on the major PLFAs in tea rhizosphere soil.

Retention time	PLFA type	Compound name	MTS	ITS	PPITS	NPITS	Feature labeling for microbes
			μg·g^−1^				
8.605	8:0	Methyl octanoate	1.21 ± 0.11ab	0.89 ± 0.01b	1.59 ± 0.24a	1.32 ± 0.18ab	
11.045	10:0	Methyl decanoate	4.35 ± 1.87b	17.95 ± 3.81a	17.43 ± 3.84a	15.41 ± 6.17ab	B
12.250	11:0	Methyl undecanoate	0.15 ± 0.02ab	0.09 ± 0b	0.24 ± 0.05a	0.20 ± 0.02a	B
13.330	12:0	Methyl dodecanoate	3.52 ± 1.79a	0.94 ± 0.09a	1.83 ± 0.29a	1.77 ± 0.13a	B
14.425	13:0	Methyl tridecanoate	0.22 ± 0.01a	0.20 ± 0a	0.25 ± 0.01a	0.25 ± 0.01a	B
15.231	14:1ω5c	Methyl myristoleate	2.41 ± 0.48a	1.19 ± 0.02b	2.08 ± 0.28ab	2.53 ± 0.27a	Oxygen addictive-GN
16.240	14:0	Methyl tetradecanoate	0.55 ± 0.22b	0.21 ± 0.04bc	0.00 ± 0c	1.56 ± 0.33a	B
16.325	15:0	Methyl pentadecanoate	0.27 ± 0.04a	0.52 ± 0.18a	0.35 ± 0.13a	0.41 ± 0.07a	B
17.075	16:0	Methyl palmitate	10.84 ± 1.45a	7.54 ± 0.16c	5.35 ± 0.62c	13.01 ± 1.45b	B, F
17.383	16:1ω5t	(Z)-Methyl hexadec-11-enoate	ND	2.99 ± 1.26	ND	ND	AMF
18.270	17:0	Methyl heptadecanoate	0.95 ± 0.02a	0.61 ± 0.15a	0.53 ± 0.07a	0.71 ± 0.03a	B
18.980	18:2ω6t,9 t	Methyl linolelaidate	1.89 ± 0.32b	1.58 ± 0.03b	1.74 ± 0.3b	3.22 ± 0.27a	Saprophytic F
19.350	18:0	Methyl stearate	35.87 ± 4.31a	12.77 ± 0.33b	12.39 ± 1.76b	27.20 ± 2.79a	Actinomycete
19.501	br18:0	Methyl isostearate	6.33 ± 4.03	ND	ND	ND	GP
19.550	18:1ω9c	Methyl elaidate	0.7 ± 0.37b	1.21 ± 0.37a	0.47 ± 0.18c	0.87 ± 0.17a	AMF
19.688	18:2ω6c,9c	Methyl linoleate	ND	2.31 ± 0.7	ND	1.32 ± 0.66	Saprophytic F
20.040	18:3ω3,6,9	Methyl linolenate	0.42 ± 0.24b	1.38 ± 0.33a	0.21 ± 0.01b	0.89 ± 0.24a	Saccharomyces
21.460	20:3ω3,6,9	Methyl cis-11,14,17-eicosatrienoate	1.11 ± 0.03b	1.13 ± 0.03ab	1.20 ± 0.03a	1.13 ± 0.01ab	Protist
21.595	20:0	Methyl arachidate	0.49 ± 0.06a	0.59 ± 0.11a	0.58 ± 0.16a	0.78 ± 0.05a	Protist
21.874	br20:0	Methyl 18-methylnonadecanoate	0.32 ± 0.19	0.66 ± 0.35	ND	0.41 ± 0.12	B
23.120	21:0	Methyl heneicosanoate	0.34 ± 0.02a	0.29 ± 0a	0.34 ± 0.02a	0.30 ± 0.02a	-
23.565	22:1ω9	Methyl erucate	2.12 ± 1.17b	5.87 ± 0.75a	2.81 ± 0.96b	3.30 ± 0.46ab	GN
23.840	22:0	Methyl behenate	0.58 ± 0.07a	0.47 ± 0.03a	0.54 ± 0.02a	0.55 ± 0.02a	Higher organisms;GN
24.303	br22:0	Methyl 20-methyl-heneicosanoate	1.47 ± 0.25	0.82 ± 0.46	ND	1.01 ± 0.03	–
24.935	23:0	Methyl tricosanoate	0.25 ± 0.09a	0.14 ± 0.07a	0.03 ± 0c	0.10 ± 0.01a	–
25.905	24:1ω9c	Methyl Nervonate	0.28 ± 0.03a	0.24 ± 0a	0.26 ± 0.02a	0.28 ± 0.01a	–
26.651	24:0	Methyl tetracosanoate	0.38 ± 0.14a	0.83 ± 0.21a	0.37 ± 0.14a	0.47 ± 0.01a	Higher organisms
27.808	3,3Me23:0	Methyl 3,3-dimethylhenicosanoate	ND	ND	0.05 ± 0	ND	–
28.916	26:0	Methyl hexacosanoate	ND	0.11 ± 0	ND	ND	–
28.950	4,8,12Me16:0	Methyl 4,8,12-trimethyltridecanoate	ND	1.35 ± 0	ND	ND	–
31.070	28:0	Methyl octacosanoate	0.26 ± 0.09	1.40 ± 0.32	ND	ND	–

MTS, rhizosphere soil of a single cultivated tea plant; ITS, rhizosphere soil of tea plant without rhizosphere barrier; PPITS, rhizosphere soil of tea plant with rhizosphere barrier; NPITS, tea rhizosphere soil by rhizosphere net partition; ND, indicated not detected. In the same row (mean ± SD, *n* = 6), there was a significant difference between the values represented by different letters (*P* < 0.05). B, bacteria; F, fungi; GP, gram positive bacteria; GN, gram negative bacteria.

### 3.2 Effect of rhizosphere intercropping on the rhizosphere microbial diversity of tea plants

**Figure 4:**
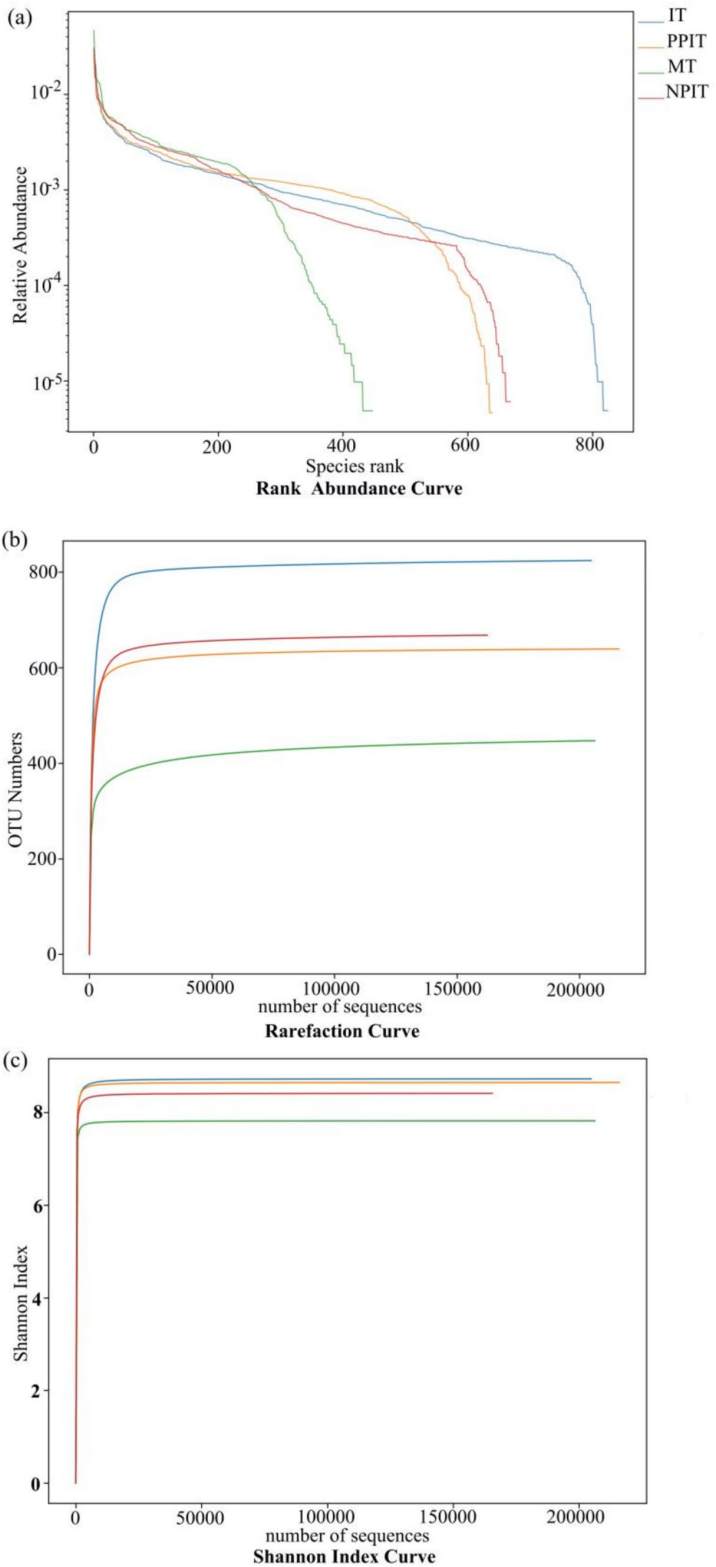
Alpha diversity analysis of soil microorganism in different cultivation patterns of tea. **(A)** Rank abundance curve, horizontal coordinates are serial numbers sorted by feature abundance, and ordinates are relative abundances of corresponding features. **(B)** Rarefaction curve, the abscissa is the number of sequencing entries extracted immediately, and the ordinate is the number of features obtained based on the number of sequencing entries. **(C)** Shannon index curve the abscissa is the number of sequencing strips randomly selected from a sample, and the ordinate is the Shannon index. The number of species found increases with the increase of sequencing amount. Until the species are saturated, increasing the number of sampling strips does not find new characteristic species.

**Figure 5:**
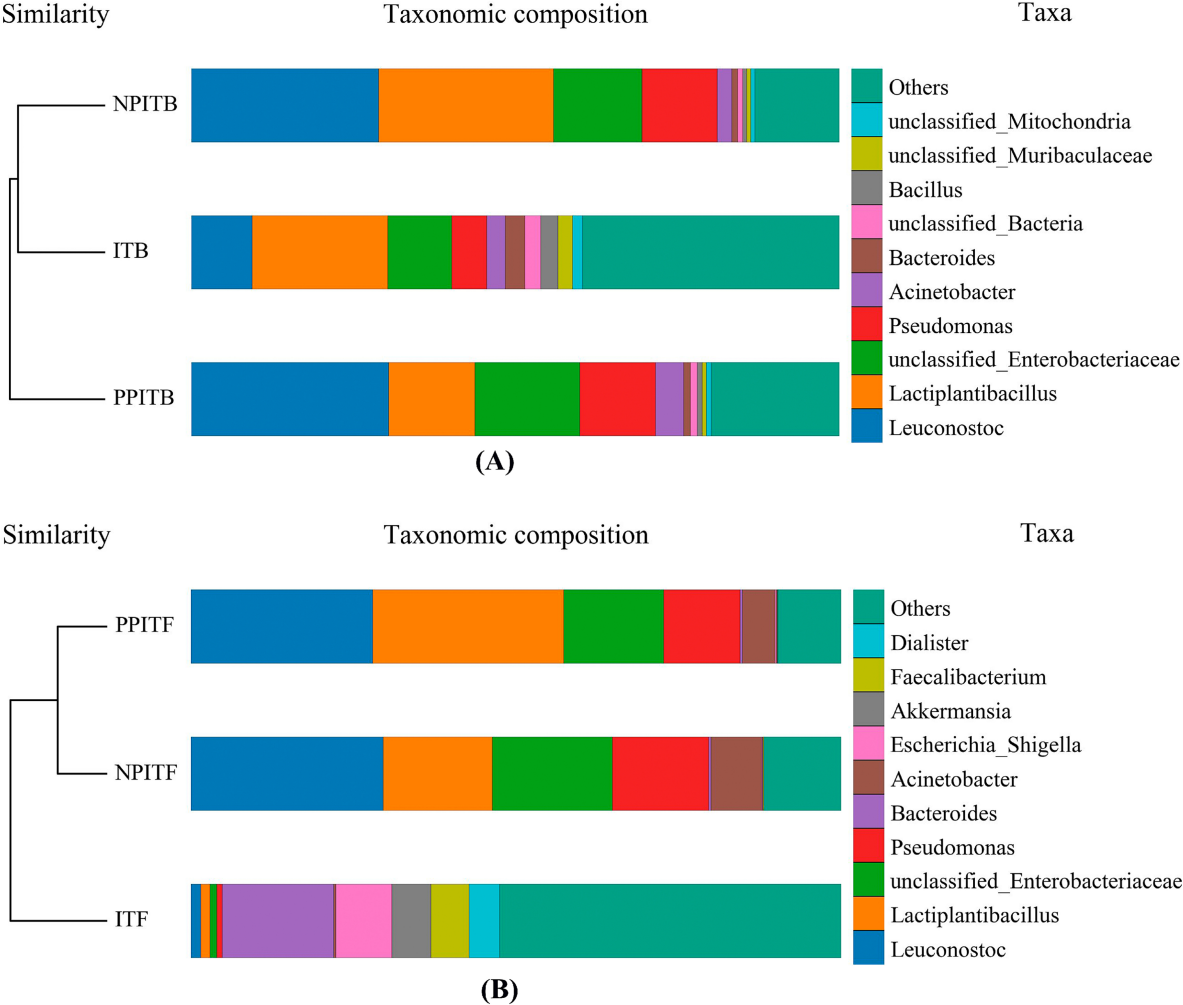
Beta diversity analysis of soil microorganism in different cultivation patterns of tea. **(A)** Cluster tree histogram combination diagram for bacteria of tea rhizosphere. **(B)** Cluster tree histogram combination diagram for fungi of tea rhizosphere. The color of the lower left image represents the color of the group where the cluster tree sample is located. The upper right image indicates the top 10 species according to the species abundance of the table, and the rest are classified as Others, unannotated objects. The species is classified as Unclassified.

At the horizontal width level, the microbial species richness of the tea rhizosphere soil under IT and NPIT was greater than that under both MT and PPIT (Figure 4A). The uniformity of the microbial species composition was also greater in the IT and NPIT treatments than in the MT and PPIT treatments, as indicated by the flatness of the curve. The dilution curve was constructed by the number of sequences and the number of species to verify that the amount of sequencing data can reflect the species diversity of the sample. The diversity of species in the soil of the IT and NPIT farming systems was greater than that in the MT and PPIT farming systems (Figure 4B). Microbial diversity was significantly greater in IT and NPIT than in MT and PPIT (Figure 4C). The microbial distribution characterized by phospholipid fatty acids and the species diversity of high-throughput sequencing reactions revealed that the intercropping of legume forages and tea plants can effectively form a stable and rich rhizosphere ecosystem. Under different intercropping modes, the bacterial species composition of tea rhizosphere under NPIT and IT intercropping conditions was closer (Figure 5A), the fungal species composition was closer under NPIT and PPIT intercropping conditions (Figure 5B), this indicated that the habitat caused by the separation had an effect on the microbial species composition in the rhizosphere of tea plants.

### 3.3 Effect of rhizosphere intercropping on differences in microbial community structure in tea rhizosphere soil

**Figure 6:**
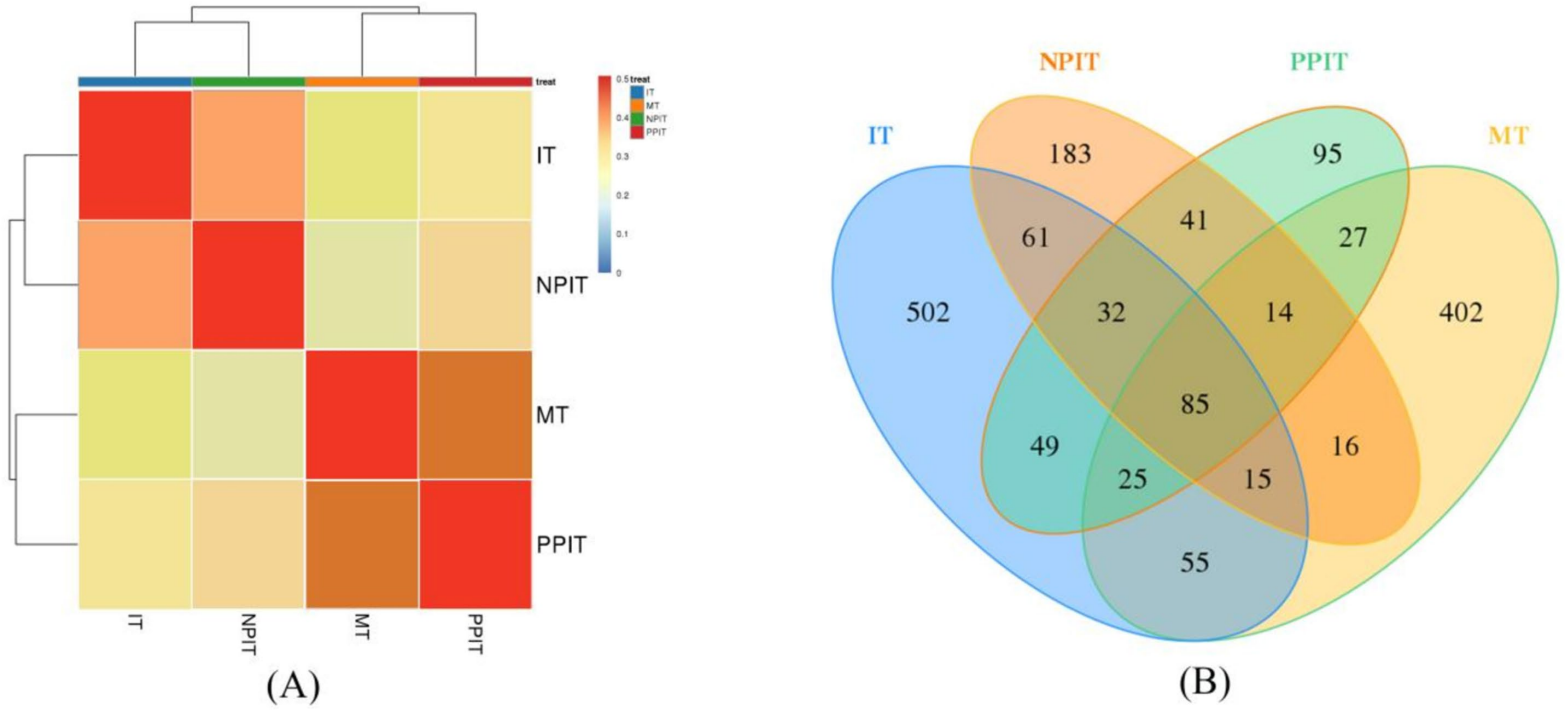
Effects of different cultivation patterns on tea rhizosphere microbial community. **(A)** Cluster analysis of different tea rhizosphere soil based on OTUs. **(B)** Venn analysis.

The rhizosphere had a significant impact on the microbial community of the rhizosphere of the tea plants (Figure 6A). Due to their similar microbial community structures, non-intercropping (IT) and net intercropping (NPIT) systems were combined at one location. The monoculture (MT) and rhizosphere intercropping (PPIT) systems were grouped together due to their more similar community structures. Venn diagram analysis also revealed that the abundances of IT, NPIT, MT and PPIT significantly differed due to the influence of the rhizosphere effect (Figure 6B). A total of 43.2% of the OTUs in the NPIT were consistent with those in the IT, and 41.0% of the OTUs in the PPIT were consistent with those in the MT. The microbial community structure of the rhizosphere of tea plants was significantly affected by the rhizosphere effect as an environmental factor.

### 3.4 Effect of rhizosphere intercropping on the ecological function of the microbial community of the tea plant rhizosphere

**Figure 7:**
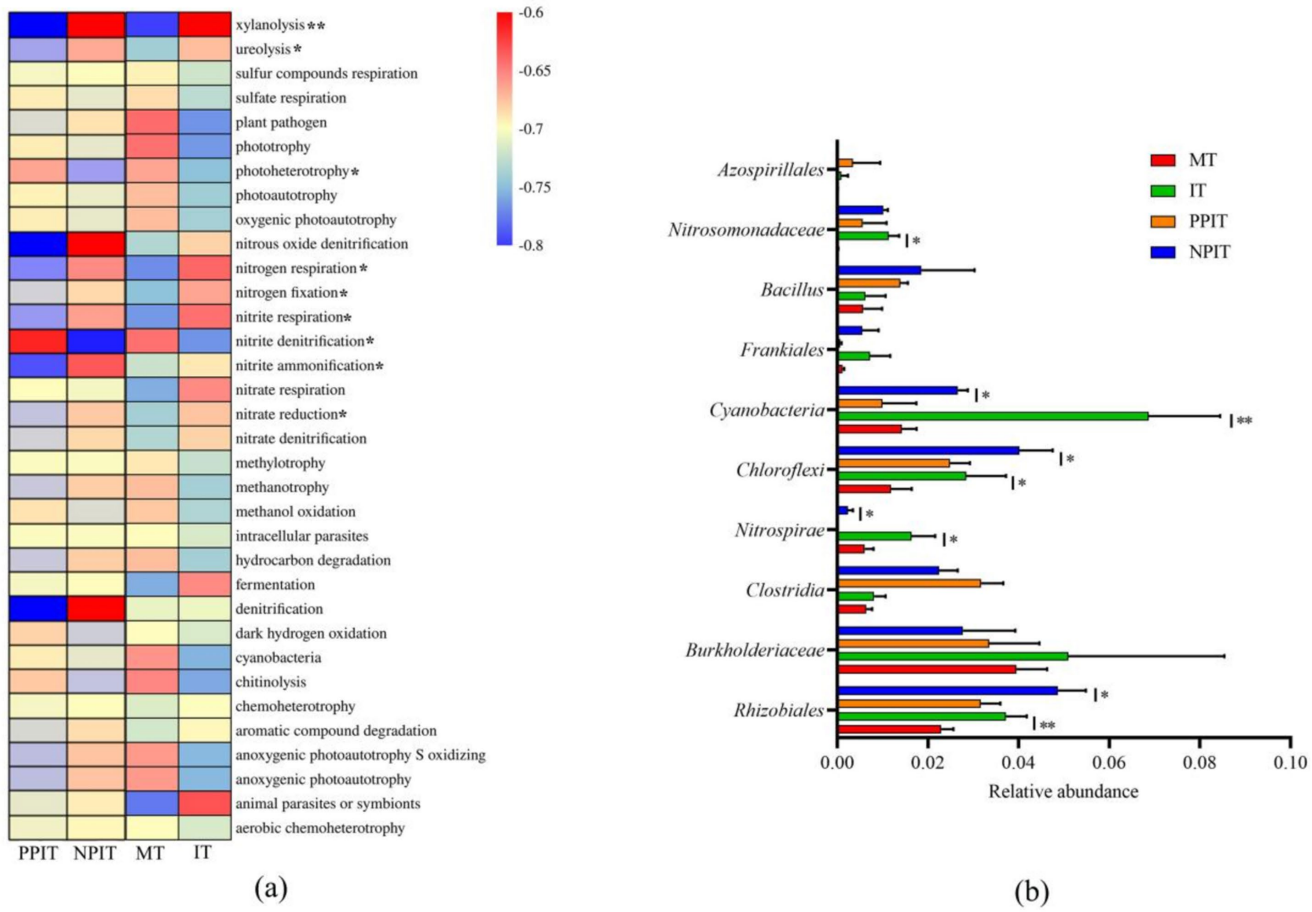
The characteristic profile of main functional microbial communities during intercropping process. **(A)** Heatmap of microbial FAPROTAX function prediction based on 16S rDNA. **(B)** The relative abundance of major functional groups related with N cycle. *Represented the same trend between PPIT vs. NPIT and MT vs. IT, and they have significant differences at a level of *p* < 0.05. *,**Showed that it has significant difference at the level of *p* < 0.05 and *p* < 0.01 in **(B)**, respectively.

A total of 34 ecological functional types were annotated in the bacterial community of the rhizosphere of the tea plant by the FAPROTAX database (Figure 7A). Compared with those in the MT and PPIT rhizospheres, the abundance of xylanolytic eco-functional bacteria in the rhizospheres of the IT and NPIT rhizospheres significantly increased by 10.1 times (*p* < 0.01). The abundance of the ecological functional groups associated with ureolysis increased significantly, by 87.0% (*p* < 0.01). There was a significant decrease of 48.7% (*p* < 0.05) in the number of ecological functional groups exhibiting photoheterotrophy. The abundances of microbial groups with ecological functions such as nitrogen respiration, nitrogen fixation, nitrite respiration, nitrite ammonification, and nitrate reduction increased by 102.7% (*p* < 0.05), 101.9% (*p* < 0.05), 145.2% (*p* < 0.05), 127.8% (*p* < 0.05), and 39.3% (*p* < 0.05), respectively. There was a 76.6% (*p* < 0.05) decrease in the abundance of the ecological functional groups associated with nitrite denitrification. *Rhizobia, burkholderia, clostridia, nitrospirae, chloroflexi*, *cyanobacteria*, *frankiales, bacillus, nitrosomonadaceae, and azospirillales* were the main microbial groups associated with the rhizosphere N cycle of tea plants (Figure 7B). Compared with those in MT and PPIT, the abundances of *rhizobium* and *nitrospira* in IT and NPIT significantly increased by 58.10% (*p* < 0.01) and 315.6% (*p* < 0.01), respectively, while those of *chloroflexi* and *cyanobacteria* increased by 100.9% (*p* < 0.05) and 275.2% (*p* < 0.05), respectively. The rhizosphere of the tea plant, which was mediated by the leguminous forage roots, had a greater amount of active decomposition of organic matter such as xylan and urea. Its action in the rhizosphere was beneficial to the availability of nutrients and to soil fertility. The rhizosphere effect of intercropping significantly affected the bacterial flora of N-cycling ecological function types, such as *rhizobia*, *nitrospirae, chloroflexi*, and *cyanobacteria*.

### 3.5 Effect of rhizosphere intercropping on different nutritional fungal community structures

**Figure 8:**
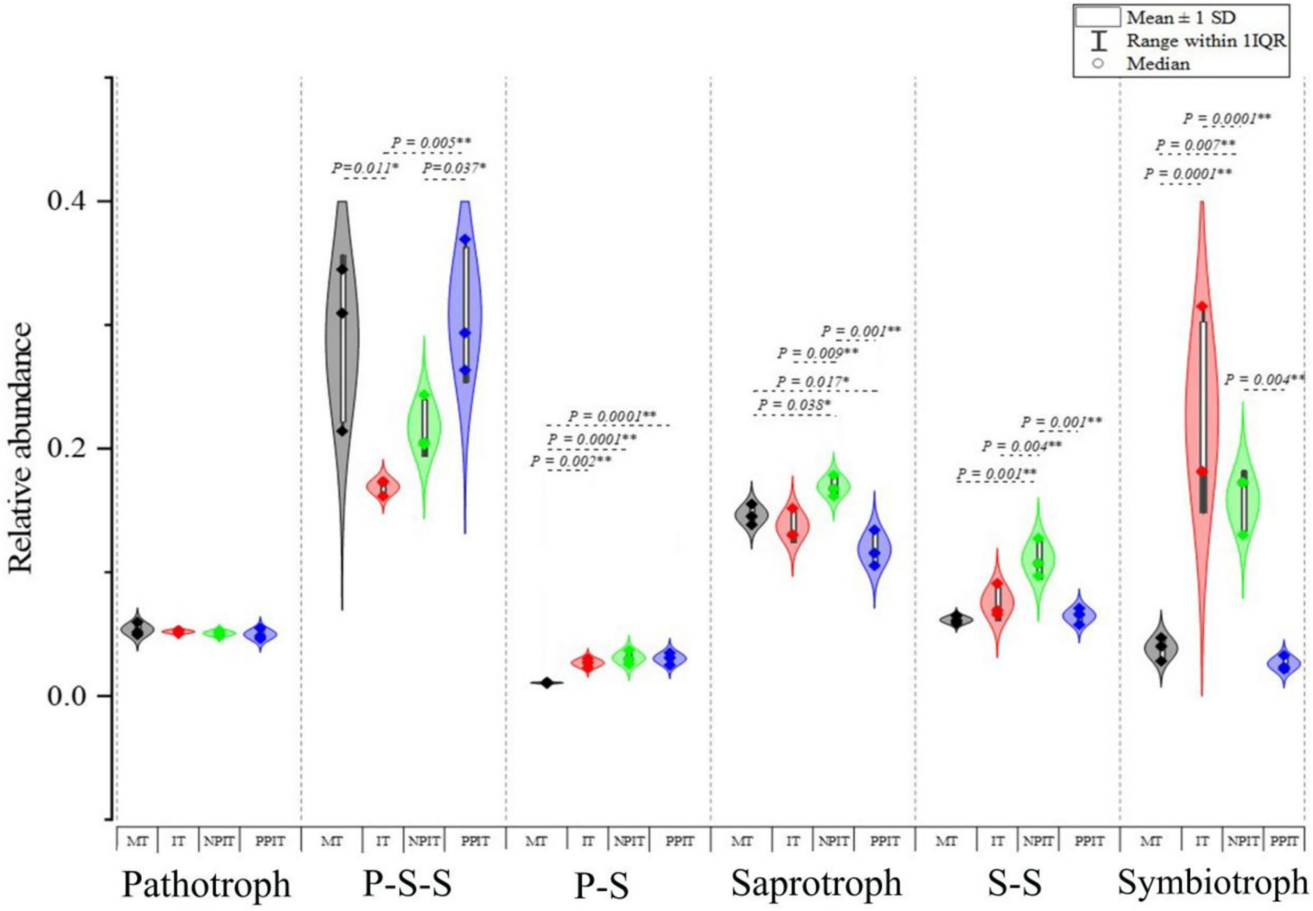
The effects of different rhizosphere intercropping on of main nutritional life type of fungal communities in tea rhizosphere soil. P-S-S, pathotroph-saprotroph-symbiotroph; P-S, pathotroph-symbiotroph; S-S, saprotroph-symbiotroph. ♦ In the chart symbols with 1st Quartile, Median, and 3rd Quartile. Range within 1IQR denotes the Inner Quartile Range, this encompasses the interval stretching from the 1st Quartile to the 3rd Quartile, augmented by one times IQR on each side. The interquartile range represents the difference between the third and first quartiles and is commonly employed to gauge the extent of data dispersion.

The functional classification of fungi in the rhizosphere of tea plants revealed six types of fungal nutritional lifestyles, namely, pathotrophic, P-S-S, P-S, saprotrophic, S-S and symbiotrophic (Figure 8). The relative abundance of pathogenic-saprophytic-symbiotic fungi in IT was 40.4% lower (*p* < 0.05) than that in MT. There was a 0.5% decrease in the relative abundance of this group of fungi in NPIT in comparison to that in PPIT. Compared with those in MT, the percentages of pathological-symbiotic and symbiotic fungi in IT increased by 2.8 and 577.4%, respectively (*p* < 0.001). Compared to those in the PPIT treatment, the abundances of saprophytic, saprophytic-symbiotic, and symbiotic fungi in the NPIT treatment increased by 19.1% (*p* < 0.01), 4.7% (*p* < 0.01), and 265.5% (*p* < 0.01), respectively. The rhizosphere process of intercropped plants significantly affects the nutritional pattern of fungi in the rhizosphere soil of tea plants, especially that of symbiotic nutritional fungi.

**Figure 9:**
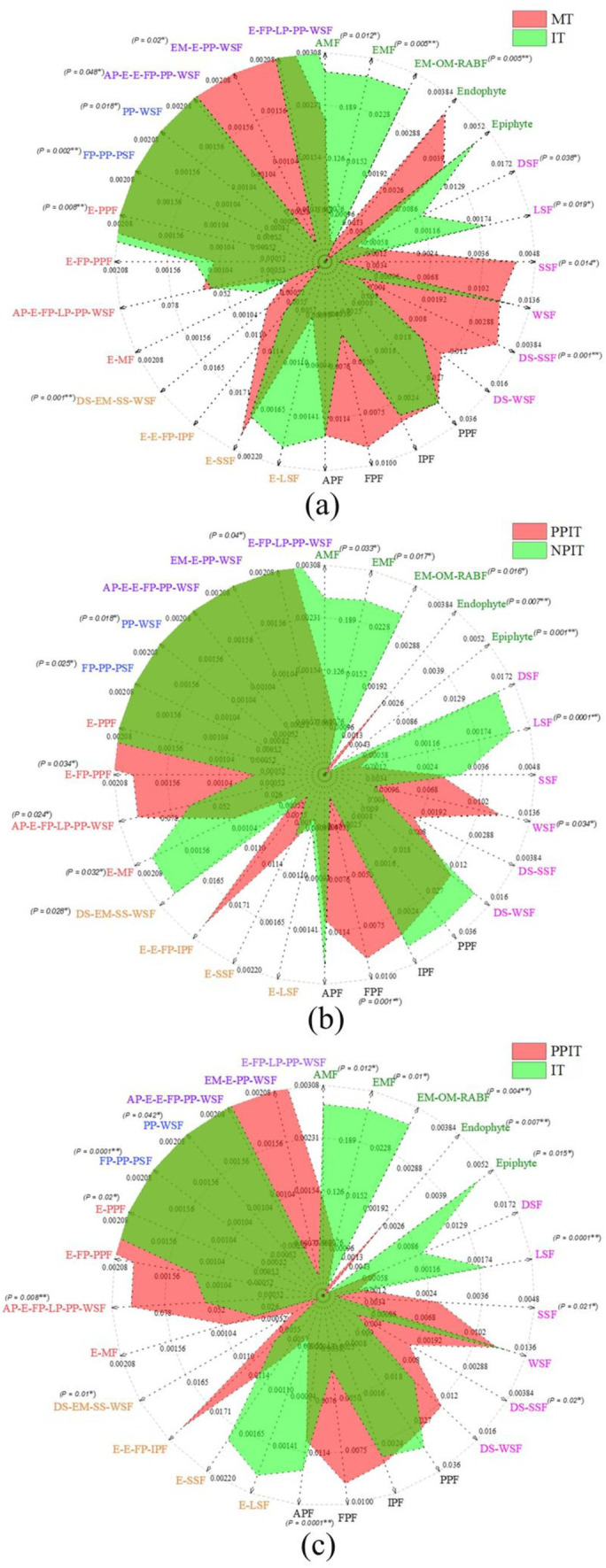
Effect of different intercropping patterns on distribution of fungi functional groups in tea plant rhizosphere soil based on FUNGuild database. **(A)** Difference of functional fungi in rhizosphere soil between MT and IT. **(B)** Difference of functional fungi in rhizosphere soil between PPIT and NPIT. **(C)** Difference of functional fungi in rhizosphere soil between PPIT and IT. These abbreviations marked different colors represent fungi with specific nutritional patterns (Table 2).

On the basis of the phenotypic prediction of tea plant fungi, the soil fungi in the rhizosphere of tea plants were classified into 28 nutritional life types (Table 2). By summarizing the relative abundance of each of the nutrient groups in the fungal community, a radar map of fungal nutrient types in the intercropping rhizosphere process showed that E-PPF, PP-WSF and PPF were the main overlapping groups of fungi in the rhizosphere of MT and IT (Figure 9A). Compared to those in MT, the abundances of E-PFP, FP-PP-PSF, and PP-WSF in IT decreased by 54.2% (*p* < 0.01), 58.9% (*p* < 0.01), and 78.7% (*p* < 0.05), respectively. The few overlapping regions of the two trophic groups were mainly in EM-E-PP-WSF, AMF, EMF, EM-OM-RAB, endophytic, DSF, LSF, SSF, DS-SSF, and FPF. In the rhizosphere of MT, the more abundant groups EM-E-PP-WSF, SSF, DS-SSF, and FPF were mainly distributed. AMF, EMF, EM-OM-RAB, DSF, and LSF were significantly distributed in the soil of the IT rhizosphere. E-PPF, FP-PP-PSF, PP-WSF, PPF and other fungal groups were also found in the overlapping fungal communities in the rhizosphere of PPIT and NPIT (Figure 9B). Compared with those in PPIT, the abundances of FP-PP-PSF and PP-WSF in NPIT were 18.3% (*p* < 0.05) and 83.5% (*p* < 0.05) lower, respectively. Fungal trophic groups such as AMF, EMF, EM-OM-RAB, DSF, and LSF were significantly distributed in the rhizosphere soil of the NPIT. E-MF and DS-EM-SS-WSF had significant distributions in the rhizosphere of NPIT. FPF, E-FP-PPF, and AP-E-FP-LP-PP-WSF were abundant in the PPIT rhizosphere soil (*p* < 0.01). The rhizosphere effect of legume forage rhizosphere secretions can effectively promote the enrichment of symbiotic mycorrhizal fungi in the rhizosphere soil of tea plants and reduce the damage of fungal pathogens to tea plant roots. Such a trend was also observed in the PPIT and IT (Figure 9C). The pathogenic-saprophytic trophic type was the main group of fungi that was significantly distributed in the rhizosphere soil of the PPIT tea plants. Mycorrhizal symbionts were significantly distributed in the IT rhizosphere. This indicated that the rhizosphere effect of tea/legume intercropping promoted the colonization of the symbiotic mycorrhizal vegetative fungal community in the tea rhizosphere soil.

**Table 2:** Summary of main fungal functional groups in tea rhizosphere soil based on fungal function prediction.*

Trophic mode	Functional guild	Abbreviation
Symbiotroph	Arbuscular mycorrhizal	AMF
	Ectomycorrhizal	EMF
	Ectomycorrhizal-Orchid mycorrhizal-Root associated biotroph	EM-OM-RABF
	Endophyte	Endophyte
	Epiphyte	Epiphyte
Saprotroph	Dung saprotroph	DSF
	Leaf saprotroph	LSF
	Soil saprotroph	SSF
	Wood saprotroph	WSF
	Dung saprotroph-Soil saprotroph	DS-SSF
	Dung saprotroph-Wood saprotroph	DS-WSF
Pathotroph	Plant pathogen	PPF
	Insect pathogen	IPF
	Fungal parasite	FPF
	Animal pathogen	APF
Saprotroph-symbiotroph	Epiphyte-Litter saprotroph	E-LSF
	Endophyte-Soil saprotroph	E-SSF
	Endophyte-Epiphyte-Fungal parasite-Insect parasite	E–E-FP-IPF
	Dung-Saprotroph-Ectomycorrhizal-Soil saprotroph-Wood saprotroph	DS-EM-SS-WSF
Pathotroph-symbiotroph	Ericoid mycorrhizal	E-MF
	Animal-pathogen-Endophyte-Fungal parasite-Lichen parasite-Plant pathogen-Wood saprotroph	AP-E-FP-LP-PP-WSF
	Endophyte-Fungal parasite-Plant pathogen	E-FP-PPF
	Endophyte-Plant pathogen	E-PPF
Pathotroph-saprotroph	Fungal parasite-Plant pathogen-Plant saprotroph	FP-PP-PSF
	Plant pathogen-Wood saprotroph	PP-WSF
Pathotroph-saprotroph-symbiotroph	Animal-Pathogen-Endophyte-Epiphyte- Fungal parasite-Plant pathogen-Wood saprotroph	AP-E–E-FP-PP-WSF
	Ectomycorrhizal-Endophyte-Plant pathogen-Wood saprotroph	EM-E-PP-WSF
	Endophyte-Fungal parasite-Lichen parasite-Plant pathogen-Wood saprotroph	E-FP-LP-PP-WSF

*This grouping of fungal phenotypes based on FUNGuild database (Nguyen et al., 2016).

### 3.6 Effects of rhizosphere intercropping on growth-promoting and pathogenic bacteria in the tea rhizosphere

**Figure 10:**
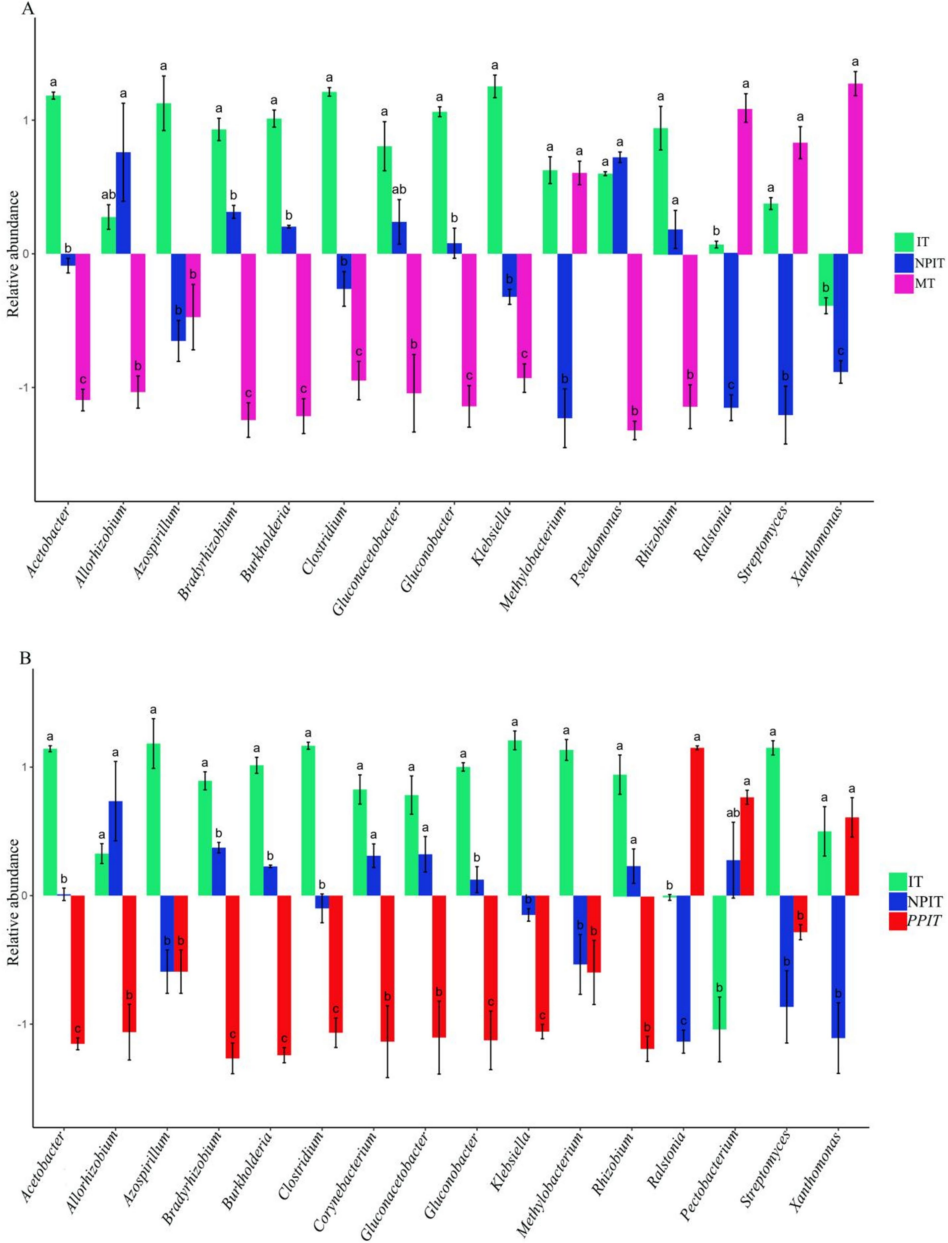
Distribution of bacteria between plant-promoting and pathogenic types in tea rhizosphere by different cultivations. **(A)** Multiple comparative analysis of MT, IT and NPIT. **(B)** Multiple comparative analysis of PPIT, IT and NPIT between plant-promoting and pathogenic bacteria. Error bars represent the standard deviation (SD) of the mean, indicating the precision of the mean distribution of bacteria across three biological replicates (*n* = 3). In the same genus, different letters indicated that there were significant differences between different treatments (*p* < 0.05), and when the bars were located on both sides of the horizontal axis, the difference trend was more obvious.

The main characteristic growth-promoting bacteria and pathogenic bacteria have been widely reported in the rhizosphere soil of crops (Supplementary Table S3). Twenty species of rhizosphere growth-promoting bacteria and four species of rhizosphere pathogenic bacteria with high relative abundances were found in the rhizosphere microbiome of tea plants. Compared with those in MT, the relative abundances of plant growth-promoting bacteria such as *allorhizobium*, *bradyrhizobium*, *rhizobium*, *burkholderia*, *gluconacetobacter*, *gluconobacter*, and *pseudomonas* were significantly greater in IT and NPIT (*p* < 0.05) (Figure 10A). Compared with those in MT, the relative abundances of *xanthomonas* and *ralstonia* decreased significantly in IT and NPIT (*p* < 0.05), by 90.2 and 80.2%, respectively (Figure 10B). Compared with those in the PPIT treatment, the relative abundances of growth-promoting bacteria such as *allorhizobium*, *bradyrhizobium*, *rhizobium*, *burkholderia*, *corynebacterium*, *gluconacetobacter*, and *gluconobacter* in the IT and NPIT treatments increased significantly, by 1.5, 12.4, 1.9, 60.3, 1.7, 3.7, and 3.0 times, respectively. Compared to those in the PPIT group, the relative abundances of pathogenic bacteria such as *xanthomonas* and *ralstonia* in the IT and NPIT groups decreased significantly, by 25.4 and 84.9%, respectively. The rhizosphere environment of intercropping systems without a barrier and with a net barrier was more conducive to the growth of growth-promoting bacteria.

**Figure 11:**
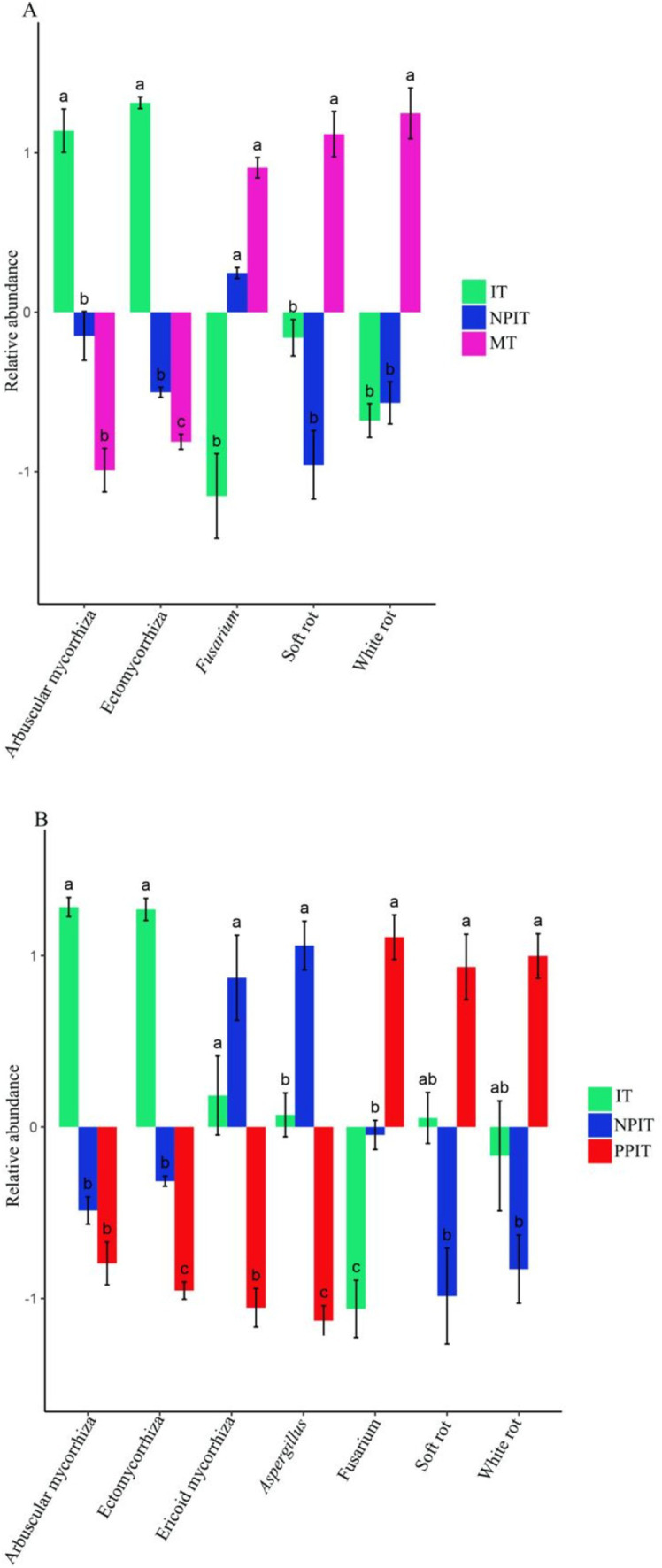
Distribution of fungi between plant-promoting and pathogenetic type in tea rhizosphere by different cultivations. **(A)** Multiple comparative analysis of rhizogenic or pathogenic fungi flora of MT, IT and NPIT. **(B)** Multiple comparative analysis of IT and NPIT and PPIT between plant-promoting and pathogenic fungi flora. Error bars represent the standard deviation (SD) of the mean, indicating the precision of the mean distribution of fungi across three biological replicates (*n* = 3), bars (mean ± SD, *n* = 3) within different letters bars showed that different treatments had significant difference at the level of *p* < 0.05.

In this study, three types of growth-promoting fungi and two types of pathogenic bacteria were found in the rhizosphere of tea plants. The growth-promoting bacteria were *aspergillus, penicillium, and trichoderma*, and the pathogenic bacteria were *fusarium* and *curvularia*. There was a significant (*p* < 0.05) increase in the abundance of ectomycorrhizal flora in the IT and NPIT treatments (Figure 11). There was a significant decrease in the abundance of fusarium pathogenic fungi in the IT and NPIT treatments (*p* < 0.05). Compared to those in MT and PPIT, pathogenic fungi such as white rot and soft rot were significantly reduced by 47.3% (*p* < 0.05) and 18.3% (*p* < 0.05) in IT and NPIT, respectively.

### 3.7 Effect of rhizosphere intercropping on GHGs in different farming systems

**Figure 12:**
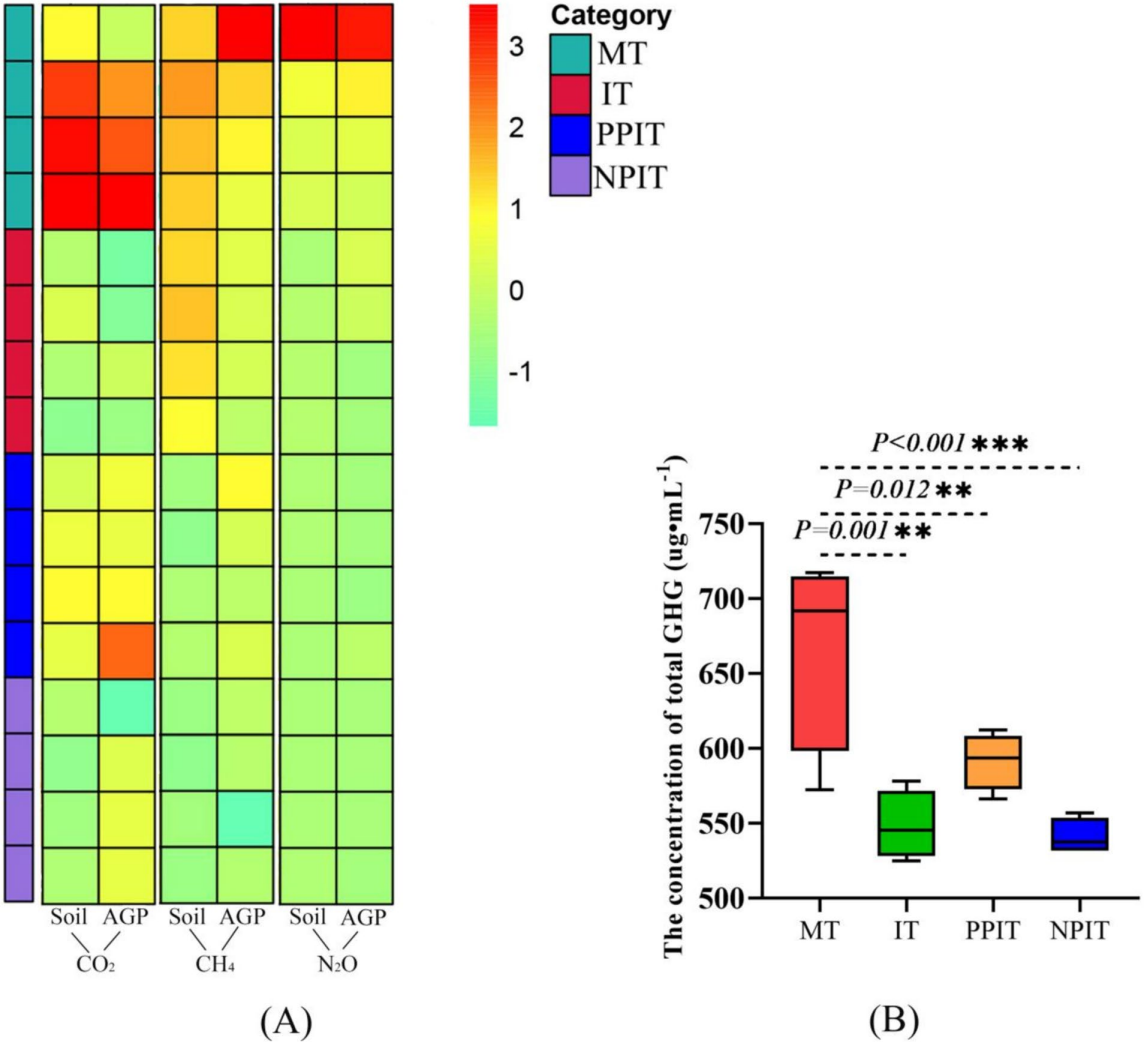
Greenhouse gas (GHG) emissions in tea - legume coculture system. **(A)** Heatmap analysis for characterization of CO_2_, CH_4_ and N_2_O from aboveground parts of plants and soils among MT and intercropping (IT, PPIT and NPIT). **(B)** Cumulative state of CO_2_, CH_4_ and N_2_O in different systems. **(A)** The same color strip on the left side of the heatmap represented the same system. The vertical level of the heatmap represented the difference in the concentration of GHG emission from the same part in the different system. The gradient of the color difference bar transitioned from a vivid green to a profound red, signifying a progressive escalation in greenhouse gas emissions. A negative value indicates reduction/increase compared to a reference. **(B)** The total emission concentration of GHGs (CO_2_, CH_4_, and N_2_O) acquired at the same time.

In different cultivation systems, CO_2_, CH_4_, and N_2_O have a variety of sources, such as the soil itself and different cultivated crops. In this study, the emission status of three GHGs from the soil and aboveground parts, including tea plants and forage legumes, was explored. In terms of concentration, CO_2_ was the most important GHG, averaging approximately 97.6%. Compared with those in MT, the aboveground and soil fractions of IT in the rhizosphere non-intercropping system decreased significantly by 27.2% (*p* < 0.01) and 37.9% (*p* < 0.001), respectively (Figure 12A). In the intercropping system, compared with those in the PPIT system, the soil CO_2_ emissions in the NPIT system decreased by 14.7% (*p* < 0.05). The average amount of CH_4_ was approximately 0.4% of the total. The emission of CH_4_ from the above-ground parts of the tea plants in the IT treatment was reduced by 11.2% (*p* < 0.05) in comparison with that in the MT treatment. The average amount of N_2_O in the air was approximately 2.0%. In comparison with those in the MT treatment, the emissions of N_2_O in the aboveground part and soil in the IT treatment decreased by 4.4 and 40.3%, respectively (*p* < 0.05). The concentrations of GHGs in the IT and NPIT plants were significantly lower than those in the MT plants (Figure 12B). Moreover, the number of GHGs in the NPIT was 8.6% lower than that in the PPIT.

## 4 Discussion

Phospholipid fatty acids (PLFAs) are the major components of nearly all living soil microbial cell membranes. PLFA technology can be used to effectively monitor the overall level of change in microorganisms in rhizosphere soil. The MRM method was used for the absolute quantification of 29 types of PLFAs biomarker in the range of C6-C24 in the rhizosphere soil of different cultivated tea plants. The rhizosphere effect produced by rhizosphere intercropping significantly increases the total amount of bacteria, fungi and AMF (Zhang et al., 2020; Gao et al., 2021). The rhizosphere effect of intercropping mediated the formation of rich and diverse rhizosphere sediment habitats in the rhizosphere soil of tea plants. It promoted the colonization of various groups of microorganisms in the rhizosphere of tea plants. In plant-microbial rhizosphere interactions, AFMs are mainly dependent on the main sources of carbon, such as fatty acids transferred from the roots of plants for symbiotic processes (Jiang et al., 2017). A large proportion of fatty acids were detected in the rhizosphere of the tea plants during the intercropping rhizosphere chemical dialog. This could be an important material basis for the colonization of the rhizosphere of tea plants by mycorrhizal vegetative fungi (Devi et al., 2020). The composition of the root exudates from both sides changed in response to intercropping. The nutritional niche of rhizosphere microbial communities was effectively expanded by different types of secreted components. Consequently, different types of microbial groups are recruited to the tea rhizosphere (Zhalnina et al., 2018). The rhizosphere treatment significantly increased the total amount of PLFAs. It was again shown that the rhizosphere effect created by intercropping tea plants had greater advantages in recruiting microorganisms.

For a better understanding of the changes in the community structure. By comparing the ratio of bacteria to fungi in the rhizosphere, it was found that in the rhizosphere (no barrier or net barrier) intercropping system, the bacteria/fungi ratio did not change significantly. This indicated that the rhizosphere effect formed during the intercropping process could better maintain the stability of the microbial community structure. In many cases, the entire habitat system of a single cultivation process is susceptible to both biotic and abiotic stresses (Pervaiz et al., 2020). An imbalance in the rhizosphere soil microbial community structure is a major cause of disasters (Hiddink et al., 2010). Microbial diversity is an important driver of the stability of microbial community structure in the rhizosphere. This process helps the microbial community remain stable when environmental factors fluctuate (García-García et al., 2019). Based on the analysis of alpha microbial diversity, the results of this study showed that the rhizosphere effect of the intercropping system played an important role in improving the microbial diversity of the tea rhizosphere. The rhizosphere effect of intercropping has important ecological implications for stabilizing microbial community structure (Liu et al., 2021). It plays an important role in maintaining the stability of the microbial ecosystem of the tea rhizosphere.

**Figure 13:**
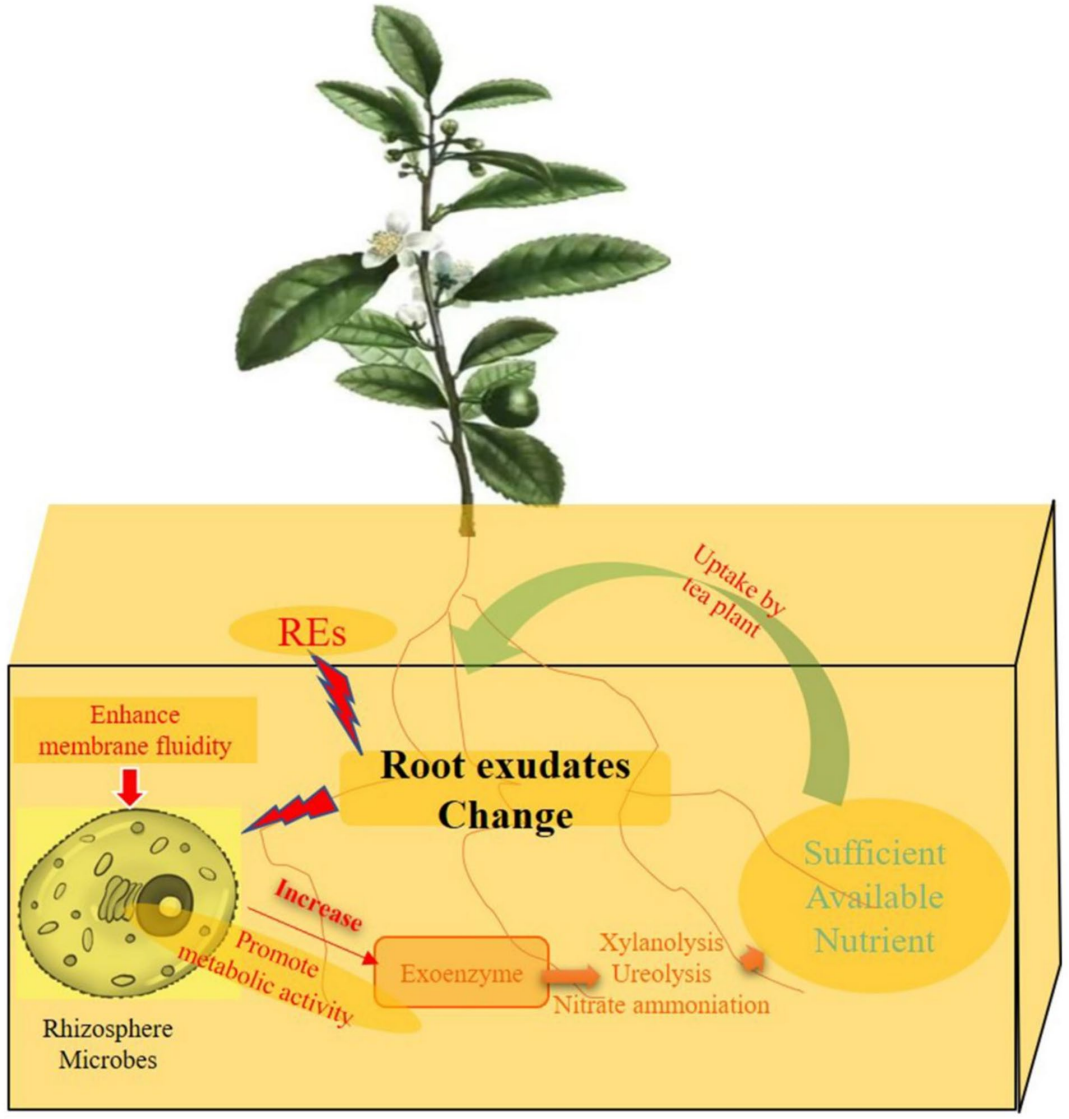
Possible formation pathway of rhizosphere priming effects of tea plant by Intercropping process. REs of tea/forage legume intercropping could be able to change the exudates secreted by tea plant root. Changes of rhizosphere environment can enhance the fluidity of cell membrane in rhizosphere microbes, and promote microbial metabolic activity. Microorganisms can secrete more exoenzymes to the tea rhizosphere, and improve the metabolic pathways, including xylanolysis, ureolysis and nitrate ammoniation process, and eventually provide sufficient available nutrients for the absorption and utilization of tea roots and promote the growth of tea plants.

The response of microorganisms to environmental stress can be directly reflected in the ratio of unsaturated to saturated fatty acids (Yao et al., 2016). This study suggested that the rhizosphere effect of intercropping increased the proportion of unsaturated fatty acids in the cell membrane phospholipids of tea rhizosphere microorganisms. This change effectively improved the fluidity of the microbial cell membrane. It plays an important role in facilitating material and energy exchange between microorganisms and the environment, and adaptation to changes in the rhizosphere environment is highly important (Harayama and Shimizu, 2020). Tea rhizosphere microorganisms can easily enhance their adaptability to the rhizosphere environment by improving their cell membrane fluidity via the rhizosphere effect. Fluidity increases the intensity of metabolic and energetic exchanges between microorganisms and the surrounding environment (Weissbrodt et al., 2020). The rhizosphere effect of intercropping was beneficial for enhancing the activity of rhizosphere microorganisms, which is highly important for the rhizosphere priming effect (Cui et al., 2023). The possible mechanism of rhizosphere priming in the process of tea tree intercropping is as follows (Figure 13). When the tea root system senses the rhizosphere effect of legume forage, microorganisms living in the rhizosphere sense the environmental changes caused by changing exudates. By increasing the fluidity of its own outer membrane, it adapts to this new rhizosphere environment. The frequent exchange of substances between microorganisms and the rhizosphere was facilitated by the increase in membrane fluidity. More extracellular enzymes are released to the outside during the exchange process (Cui et al., 2023; Van Hamme et al., 2006). Soil extracellular enzymes promote the decomposition of rhizosphere organic matter so that more effective nutrients are absorbed and utilized by tea plants, thereby promoting the growth of tea plants (Brzostek et al., 2013).

**Figure 14:**
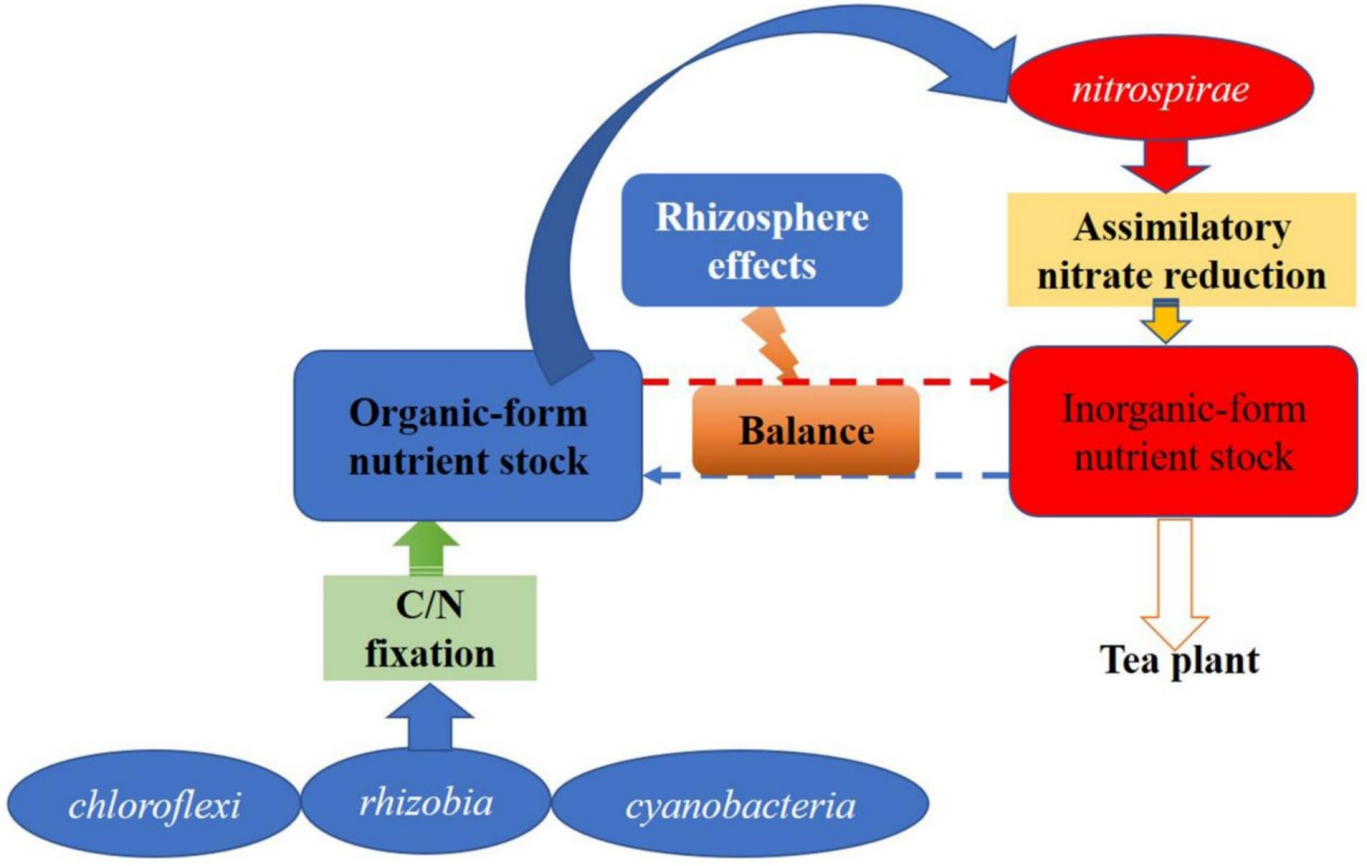
Possible pathways of rhizosphere effects to maintain nutrient balance of tea plant in intercropping process. Tea roots uptake nutrient (NH_4_^+^) from the inorganic nutrient stock. When the inorganic nutrient stock is insufficient, the assimilatory nitrate reduction process of *nitrospirae is* initiated by the rhizosphere effects, and the rhizosphere organic nutrient stock is transformed into inorganic nutrient to recharge the inorganic nutrient stock. When the organic nutrient stock is insufficient, the C/N fixation of microbes represented by rhizobia, *cyanobacteria* and *chloroflexi* was initiated to supply the organic nutrient stock, and maintain the nutrient balance of tea rhizosphere.

Tea-legume intercropping is a healthy and sustainable rhizosphere process. In this study, phenotypic annotations such as FAPROTAX ecological function and FUNGuild nutritional life type were performed on microbial communities. Biogeochemical processes such as the decomposition of organic matter such as xylan and urea and the reduction of assimilative nitrate are significantly enhanced by the rhizosphere effect (Enebe and Babalola, 2021). The enhanced decomposition of organic matter may be mainly due to the rhizosphere priming effect triggered by the increased fluidity of the rhizosphere microbial membrane (Khan et al., 2021). This organic matter decomposition and effective nutrient formation pathway may be derived from xylan-urea decomposition and assimilative nitrate reduction (Nivethadevi et al., 2021). The bacterial communities, especially those of *rhizobia*, *Nitrospira, Chloroflexi*, and *cyanobacteria*, which characterize the biological functions of soil nutrient cycling, were abundant in the rhizosphere of tea plants. We assumed that the enhanced decomposition of organic matter such as organic carbon and nitrogen in the rhizosphere could increase the source of inorganic nutrients in the rhizosphere of tea plants to some extent (Dijkstra et al., 2013). At the same time, the bacteria in the C and N fixation process synthesize their own substances to enrich the rhizosphere organic nutrient pool source of microbial biomass C and N (Pii et al., 2015). The most important contributors to the enrichment of this organic matter are likely to be the ecologically functional bacteria listed above (Logue et al., 2016). Moreover, when the supply of inorganic nutrients in the rhizosphere of tea plants is insufficient, organic nutrients can be a source of available nutrients (NH_4_^+^-N and NO_3_^−^-N) to tea plants through the assimilatory nitrate reduction pathway of functional flora. The inorganic and organic nutrient pools of the rhizosphere could maintain a good dynamic equilibrium process. That is, the rhizosphere effect formed during the intercropping process also plays an important role in maintaining the balance of nutrients in the rhizosphere of tea plants (Figure 14).

The colonization of the tea rhizosphere by AMF can be improved by the rhizosphere effect. This may be due to the corelease of flavonoids from legumes and fatty acids from tea roots. The secretion of a large number of flavonoids in roots can effectively enhance the colonization of arbuscular mycorrhizal fungi in the rhizosphere of host plants (Tian et al., 2021). The fungal flora with mycorrhizal symbiosis as the nutritional mode tended to occur in the rhizosphere intercropping system, particularly the species of fungi in which the ectomycorrhizal and arbuscular mycorrhizal symbioses were nutritionally important. ECM and AMF can promote the stability of soil aggregate structure through physical processes such as entanglement and net trapping (Courty et al., 2010; Jeewani et al., 2021). The fixation of organic matter is based on stable soil aggregates, which may be due to the extensive colonization of ectomycorrhizal fungi in the rhizosphere of tea plants (Parihar et al., 2020; Bag et al., 2022). The intercropping of legumes and tea plants can significantly increase the biomass of mycorrhizal fungi in the rhizosphere of tea plants (Li et al., 2022). Mycorrhizal fungi can expand the space for roots to absorb nutrients, which enhances the ability of tea plants to absorb nutrients from outside of the root zone (Ortaş and Rafique, 2017). This approach provides a viable solution to the problems of nutrient leaching and the migration of unstable nutrients caused by acidification of the soil.

The rhizosphere effect formed during the intercropping process was beneficial to the enhancement of plant growth-promoting bacteria, including *allorhizobium*, *bradyrhizobium*, *rhizobium*, *burkholderia*, *gluconacetobacter*, and *gluconobacter*. Plant growth-promoting bacteria not only play an important role in providing plants with sufficient nutrients but also play an important role in the biological control of plant pathogens (Hayat et al., 2010). Rhizosphere growth-promoting bacteria can produce antibiotics, hydrolases, or specific substances that inhibit the growth of pathogenic bacteria (Xiao et al., 2016; Ma et al., 2015). The rhizosphere of tea plants can fix and release abundant carbon sources such as fatty acids during the rhizosphere process of intercropping. This allowed mycorrhizal fungi and other growth-promoting bacteria to colonize. Moreover, plant growth-promoting bacteria have biological prevention and control effects, so the colonization process of pathogens in the rhizosphere of tea plants is not dominant, which effectively reduces the risk of pathogen outbreaks in the rhizosphere of plants (Glick, 2012; Kong and Liu, 2022).

From the above results, it was not difficult to determine that the rhizosphere significantly affects the number of microorganisms involved in the ecological functions of N cycling and the number of mycorrhizal symbiotic nutrients in the rhizosphere of tea plants. Nitrogen-fixing bacteria, *cyanobacteria*, *chloroflexi* and nitrifying bacteria are the main functional groups in N-cycling ecosystems and play important roles in soil C and N fixation (Xian et al., 2020; Kang and Jia, 2019; Liu, 2022). AMF play an important role in reducing N_2_O during the N cycle. Soil nitrogen levels can be reduced by increasing the uptake of soil nitrogen by host plants and microorganisms, thereby achieving the goal of reducing nitrogen emissions (Shen and Zhu, 2021). In the process of symbiosis with host plants, AMF can convert plant photosynthates into refractory organic matter, and their own release of glomalin can promote the sequestration of carbon in soil aggregates (Parihar et al., 2020; Clemmensen et al., 2015). There was a significant downward trend in the soil CO_2_ emissions of the intercropping system mediated by the rhizosphere effect, and the overall level of greenhouse gases in the system decreased as well. The rhizosphere impact of tea-soybean intercropping is instrumental in enhancing the rhizospheric wellbeing of tea plants. It enriches and diversifies the nutrient profile in the rhizosphere, while also stabilizing the soil aggregate structure, thereby offering a substantial material and habitat foundation for the variety and recruitment of microbial communities capable of carbon sequestration. Therefore, the rhizosphere effect can promote the settlement of microbes with C and N ecological functions in tea rhizosphere soil and achieve the goal of reducing carbon emissions.

## 5 Conclusion

The tea rhizosphere, fostered through the practice of tea and legume intercropping, constitutes a robust and sustainable ecosystem. Drawing upon the rhizosphere microbial strategy, the alterations in the diversity and composition of the rhizosphere sediment surrounding tea plants have a profound impact on various facets of the microbial community within this niche. These modifications are manifest in the enhanced microbial abundance, as indicated by the increased total phospholipid fatty acid content, the elevated membrane fluidity of microbial cells due to shifts in the proportion of unsaturated fatty acids within phospholipids, the enriched species diversity, the improved nutritional optimization of the microbial flora, and the maintained dynamic equilibrium between inorganic and organic nutrients. The rhizosphere effect significantly enhanced the colonization of fungal communities, particularly in the symbiotic feeding modes of mycorrhizae and ectomycorrhizae, as well as fostered the establishment of pivotal plant growth-promoting bacteria, including *heterorhizobium*, *bradyrhizobium*, *rhizobium*, *burkholderia*, *gluconacetobacter*, and *gluconobacter*, within the rhizospheric region of tea plants. The predominance of these plant growth-promoting bacteria in the tea rhizosphere effectively curtailed the proliferation of pathogenic fungi, such as plant-pathogenic and saprophytic fungi affecting plants and wood, along with pathogenic bacteria like *xanthomonas* and *ralstonia solanacearum*, thereby contributing to a healthier tea rhizosphere environment. The rhizospheric impact generated through tea and legume intercropping facilitates the optimization of the functional flora structure in the tea rhizosphere, playing a pivotal role in the biological regulation of rhizospheric pathogens. Furthermore, this investigation has elucidated that the collaborative dynamics of carbon (C), nitrogen (N), arbuscular mycorrhizal fungi (AMF), photoautotrophic bacteria, and nitrogen-fixing bacteria within the intercropping rhizosphere can significantly mitigate soil carbon release and total greenhouse gas emissions. These findings are instrumental in enhancing our comprehension of the primary practical implications of rhizosphere intercropping in optimizing the structure of the rhizosphere community and in alleviating the impact of greenhouse gases on agricultural lands.

## Data Availability

The data presented in the study are deposited in the Sequence Read Archive (SRA), accession number PRJNA1179330.

## Footnotes

^1^ www.bioinformatics.com.cn
