# Supplementary material for: Effects of intercropping with legume forage on the rhizosphere microbial community structure of tea plants
Source: Front Microbiol. 2024 Nov 25;15:1474941. doi: 10.3389/fmicb.2024.1474941 (PMC11625550; doi:10.3389/fmicb.2024.1474941)
Supplement: Supplementary file 1 [file Table_1.docx]

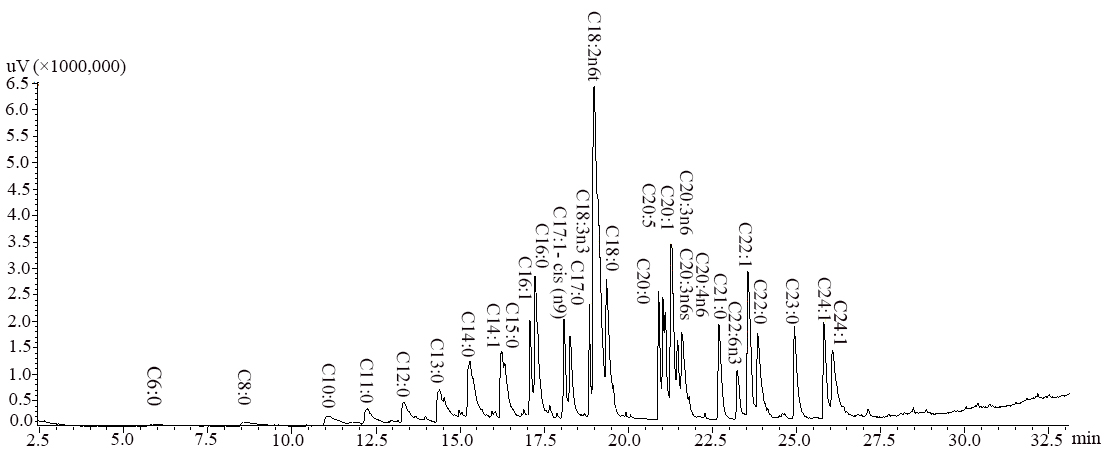


**Fig. S1** TIC of 5 µg·mL-1fatty acid methyl ester mixed standard solution using Q3 full-scan mode

Note: C6:0_methyl hexanoate; C8:0_methyl octanoate; C10:0_methyl decanoate; C11:0_methyl undecanoate; C12:0_methyl dodecanoate; C13:0_methyl tridecanoate; C14:0_methyl myristoleate; C14:1_methyl tetradecanoate; C15:0_methyl pentadecanoate; C16:0_methyl palmitate; C16:1_methyl palmitoleate; C17:0_methyl heptadecanoate; C17:1-cis(n9)_methyl cis-9-heptadecanoate; C18:0_methyl stearate; C18:2n6t_methyl linoelaidate; C18:3n3_methyl linolenate; C20:0_methyl arachidate; C20:1_methyl cis-11-eicosadienoate; C20:5_methyl cis-5,8,11,14,17-Eicosadienoate; C20:3n6_methyl cis-8,11,14-eicosatrienoate; C20:3n6s_methyl 11,14,17-icosatrienoate; C20:4n6_methyl cis-5,8,11,14-Eicosadienoate; C21:0_methyl heineicosanoate; C22:0_methyl behenate; C22:1_methyl cis-13-Docosatetraenoate; C22:6n3_DHA methyl eater; C23:0_methyl tricosanoate; C24:0_methyl Tetracosanoate; C24:1_methyl cis-15-tetracoseno

| **Table S1**. Summary of fatty acid methyl ester (FAME) product ions | | | | | | |
| --- | --- | --- | --- | --- | --- | --- |
| Components | Retention | CAS number | Qualitative ion pair ch1 | Qualitative ion pair ch2 | Qualitative ion pair ch3 | Qualitative ion pair ch4 |
| C6:0 | 5.920 | 106-70-7 | 74.00>55.10 | 87.00>55.10 | 59.00>57.10 | 74.00>71.10 |
| C8:0 | 8.605 | 111-11-5 | 74.00>55.10 | 87.00>55.10 | 87.00>59.10 | 87.00>57.10 |
| C10:0 | 11.045 | 110-42-9 | 74.00>55.10 | 87.00>55.10 | 74.00>69.10 | 87.00>83.10 |
| C11:0 | 12.250 | 1731-86-8 | 74.00>55.10 | 87.00>55.10 | 74.00>69.10 | 87.00>59.10 |
| C12:0 | 13.330 | 111-82-0 | 74.00>55.10 | 87.00>55.10 | 74.00>69.10 | 87.00>69.10 |
| C13:0 | 14.385 | 1731-88-0 | 74.00>55.10 | 87.00>55.00 | 74.00>69.10 | 87.00>83.00 |
| C14:0 | 15.290 | 56219-06-8 | 74.00>55.10 | 87.00>55.10 | 74.00>69.10 | 87.00>83.20 |
| C14:1 | 16.240 | 124-10-7 | 74.00>55.10 | 87.00>55.10 | 87.00>67.10 | 87.00>83.10 |
| C15:0 | 16.325 | 7132-64-1 | 74.00>55.10 | 75.00>55.10 | 87.00>55.10 | 74.00>69.10 |
| C16:0 | 17.075 | 1120-25-8 | 74.00>55.10 | 69.00>55.10 | 69.00>67.10 | 74.00>67.10 |
| C16:1 | 17.230 | 112-39-0 | 74.00>55.10 | 87.00>55.10 | 87.00>83.10 | 74.00>69.10 |
| C17:0 | 18.085 | 75190-82-8 | 74.00>55.10 | 69.00>55.10 | 69.00>67.10 | 74.00>69.10 |
| C17:1-*cis*(n9) | 18.270 | 1731-92-6 | 74.00>55.10 | 87.00>55.10 | 87.00>69.10 | 87.00>83.20 |
| C18:0 | 18.840 | 301-00-8 | 79.00>77.10 | 80.00>77.10 | 79.00>55.10 | 80.00>55.00 |
| C18:2n6*t* | 18.980 | 2566-97-4 | 81.00>55.10 | 81.00>67.10 | 81.00>79.10 | 67.00>55.10 |
| C18:3n3 | 19.350 | 112-61-8 | 74.00>55.00 | 87.00>55.10 | 87.00>83.10 | 74.00>69.10 |
| C20:0 | 20.900 | 2566-89-4 | 79.00>77.10 | 67.00>65.10 | 79.00>51.00 | 91.00>79.10 |
| C20:1 | 21.025 | 2734-47-6 | 79.00>77.10 | 91.00>79.10 | 91.00>77.10 | 79.00>51.10 |
| C20:5 | 21.095 | 179215 | 79.00>77.10 | 80.00>77.10 | 80.00>51.10 | 79.00>51.10 |
| C20:3n6 | 21.275 | 17364-32-8 | 81.00>67.10 | 81.00>55.10 | 67.00>55.10 | 81.00>79.10 |
| C20:3n6*s* | 21.460 | 55682-88-7 | 79.00>77.10 | 79.00>67.10 | 79.00>55.10 | 67.00>65.10 |
| C20:4n6 | 21.595 | 1120-28-1 | 74.00>55.10 | 87.00>55.10 | 74.00>69.10 | 87.00>83.10 |
| C21:0 | 22.690 | 6064-90-0 | 74.00>55.10 | 87.00>55.10 | 74.00>69.10 | 87.00>83.10 |
| C22:0 | 23.240 | 301-01-9 | 79.00>77.10 | 91.00>77.10 | 91.00>79.10 | 79.00>65.10 |
| C22:1 | 23.565 | 1120-34-9 | 69.00>67.10 | 69.00>55.10 | 67.00>55.10 | 69.00>53.10 |
| C22:6n3 | 23.840 | 929-77-1 | 74.00>55.10 | 87.00>55.10 | 74.00>69.10 | 87.00>83.10 |
| C23:0 | 24.935 | 2433-97-8 | 74.00>55.10 | 87.00>55.10 | 74.00>69.10 | 87.00>83.20 |
| C24:0 | 25.810 | 2733-88-2 | 74.00>55.10 | 69.00>55.10 | 69.00>67.10 | 74.00>69.10 |
| C24:1 | 26.070 | 2442-49-1 | 74.00>55.10 | 87.00>55.10 | 57.00>55.10 | 87.00>83.10 |

| **Table S2**. Linear equation of fatty acid methyl esters by calibration | | | |
| --- | --- | --- | --- |
| ID# | Component name | Curve equation calibration standard | Correlation |
| 1 | Methyl hexanoate | y=68963x+94870 | R^2^=0.9997 |
| 2 | Methyl octanoate | y=160611x-12784 | R^2^=0.9992 |
| 3 | Methyl decanoate | y=375543x-21194 | R^2^=0.9978 |
| 4 | Methyl undecanoate | y=591709x+16416 | R^2^=0.978 |
| 5 | Methyl dodecanoate | y=250116x-195137 | R^2^=0.9907 |
| 6 | Methyl tridecanoate | y=318426x-126402 | R^2^=0.9932 |
| 7 | Methyl myristoleate | y=82000x-19687 | R^2^=0.9382 |
| 8 | Methyl tetradecanoate | y=197690x+503692 | R^2^=0.9399 |
| 9 | Methyl pentadecanoate | y=182939x+24988 | R^2^=0.9592 |
| 10 | Methyl palmitoleate | y=47009x+17990 | R^2^=0.9512 |
| 11 | Methyl hexadecanoate | y=505393x-436683 | R^2^=0.9853 |
| 12 | Methyl 9-heptadecenoate | y=93244x+4068.3 | R^2^=0.9989 |
| 13 | Methyl heptadecanoate | y=547185x-344716 | R^2^=0.9824 |
| 14 | Methyl Linolenate | y=26172x-7075.7 | R^2^=0.9801 |
| 15 | Methyl Linoelaidate | y=61289x-20880 | R^2^=0.9451 |
| 16 | Methyl Octadecanoate | y=163641x-122774 | R^2^=0.9626 |
| 17 | Methyl Arachidonate | y=233306x-279088 | R^2^=0.8842 |
| 18 | Methyl Eicosapentaenoate | y=180041x-135260 | R^2^=0.953 |
| 19 | Methyl cis-11-eicosatrienoate | y=1000000x-1000000 | R^2^=0.9943 |
| 20 | Lineoleoyl chloride | y=114485x-112224 | R^2^=0.9562 |
| 21 | Methyl 11,14,17-icosatrienoate | y=308184x-303481 | R^2^=0.9703 |
| 22 | Methyl Eicosanoate | y=391574x-308025 | R^2^=0.9726 |
| 23 | Methyl Heneicosanoate | y=760281x-399128 | R^2^=0.9789 |
| 24 | Methyl Docosahexaenoate | y=760281x-399129 | R^2^=0.9790 |
| 25 | Methyl Erucate | y=9799x-2421.7 | R^2^=0.9898 |
| 26 | Methyl Docosanoate | y=948615x-959320 | R^2^=0.9804 |
| 27 | Methyl Tricosanoate | y=101678x+410474 | R^2^=0.997 |
| 28 | Methyl Nervonate | y=270097x-134705 | R^2^=0.9976 |
| 29 | Methyl Tetracosanoate | y=137114x+488061 | R^2^=0.9932 |
|  |  |  |  |

| **Table S3** Summary of main probiotics and pathogenic microorganisms within crop roots | | | | |
| --- | --- | --- | --- | --- |
| Types | Category | Attribution of characteristic microbia | Effects on plants | Reference |
| Plant growth-promoting microorganisms (PGPM) | Ectomycorrhiza | *basidiomycotina, ascomycotina, zygomycota, pisolithus* | nutritive symbiosis | [1] |
|  | Arbuscular mycorrhiza | *glomus* | nutritive symbiosis | [2] |
|  | Ericoid mycorrhiza | *ascomycetes: leotiales* | nutritive symbiosis | [3] |
|  | Orchid mycorrhiza | *pezizella, oidiodendron, myxotrichium, gymnascella* | nutritive symbiosis | [4] |
|  | Monotropoid mycorrhiza | *ascomycetes, basidiomycetes* | nutritive symbiosis | [5] |
|  | Arbutoid mycorrhiza | *basidiomycetes* | nutritive symbiosis | [6] |
|  | Plant growth-promoting fungi (PGPF)Fungi | *aspergillus, penicillium* | P-solubilization | [7] |
|  |  | *trichoderma* | Biocontrol | [8] |
|  | plant growth-promoting rhizobacteria (PGPR) | *rhizobium, bradyrhizobium, burkholderia, mesorhizobium, sinorhizobium, azorhizobium, allorhizobium, azospirillum, azotobacter,azoarcus,herbaspirillum,gluconacetobacter diazotrophicus,acetobacter, gluconabacter, gluconacetobacter, acidomonas, klebsiella, methylobacterium, paenibacillus, pseudomonas, rhodopseudomonas, stenotrophomonas* | N-fixation, P-solubilization, Biocontrol | [7], [9], [10], [11], [12], [13], [14], [15], [16], [17], [18], [19] |
|  |  | *frankia, alcaligenes* | N-fixation, P-solubilization | [20], [21] |
|  |  | *bacillus, curtobacterium* | P-solubilization, Biocontrol | [8], [16] |
|  |  | *clostridium, comamonas, cupravidus (ralstonia)* | N-fixation | [22], [23], [24] |
|  |  | *klebsiella, enterobacter, citrobacter, flavobacterium, agrobacterium, micrococcus, aerobacter, erwinia, R. leguminosarum* | P-solubilization | [25], [26], [27], [28], [29], [30], [31], [32] |
|  |  | *corynebacterium* | Biocontrol | [16] |
| Plant pathogenic microorganism | pathogenic fungi | *fusarium, rhizoctonia, pythium, macrophomina, colleotrichum, curvularia, botrytis,* *sclerotinia, helicobasidium mompa,* *verticillium,* *phytomyxea,* *plasmodiophora,* *spongospora,* *phytophthora，**pellicularia* | pathopoiesis | [32] |
|  | pathogenic bacteria | *clostridium botulinum, erwinia carotovora,* *agrobacterium tumefaciens, streptomyces scabies, xanthomonas,* *ralstonia,* *pectobacterium,* *streptomyces* | pathopoiesis | [30] |
| *Note：*Biocontrol indicates that microbe can inhibit plant pathogens | | | | |

[1] Tedersoo, L.; Bahram, M.; Zobel, M., How mycorrhizal associations drive plant population and community biology. Science 2020, 367, (6480), eaba1223.

[2] van der Heijden, M. G.; Martin, F. M.; Selosse, M. A.; Sanders, I. R., Mycorrhizal ecology and evolution: the past, the present, and the future. New phytologist 2015, 205, (4), 1406-1423.

[3] Smith; Sally, E., Mycorrhizal Symbiosis || Ericoid mycorrhizas. 1997, 323-346.

[4] McCormick, M. K.; Whigham, D. F.; Canchani-Viruet, A., Mycorrhizal fungi affect orchid distribution and population dynamics. New Phytologist 2018, 219, (4), 1207-1215.

[5] Lewis; J., D., Encyclopedia of Evolutionary Biology || Mycorrhizal Fungi, Evolution and Diversification of. 2016, 94-99.

[6] Johnson, N. C.; Gehring, C. A., Mycorrhizas: symbiotic mediators of rhizosphere and ecosystem processes. The rhizosphere 2007, 73-100.

[7] Reddy, C. A.; Saravanan, R. S., Polymicrobial multi-functional approach for enhancement of crop productivity. Advances in applied microbiology 2013, 82, 53-113.

[8] Zamioudis, C.; Pieterse, C. M., Modulation of host immunity by beneficial microbes. Molecular Plant-Microbe Interactions 2012, 25, (2), 139-150.

[9] Marschner, H., Marschner's mineral nutrition of higher plants. Academic press: 2011.

[10] Vandamme, P.; Goris, J.; Chen, W.-M.; De Vos, P.; Willems, A., *Burkholderia tuberum* sp. nov. and Burkholderia phymatum sp. nov., nodulate the roots of tropical legumes. Systematic and applied microbiology 2002, 25, (4), 507-512.

[11] Morgan, J.; Bending, G.; White, P., Biological costs and benefits to plant-microbe interactions in the rhizosphere. Journal of experimental botany 2005, 56, (417), 1729-1739.

[12] James, E. K.; Gyaneshwar, P.; Barraquio, W. L.; Mathan, N.; Ladha, J. K., Endophytic diazotrophs associated with rice. The quest for nitrogen fixation in rice 2000, 119-140.

[13] Ladha, J. K.; Reddy, P. M. The quest for nitrogen fixation in rice[C]. Quest for Nitrogen Fixation in Rice. 2000.

[14] Amaresan, N.; Kumar, M. S.; Annapurna, K.; Kumar, K.; Sankaranarayanan, A., Beneficial microbes in agro-ecology: bacteria and fungi[M]. Academic Press, 2020.

[15] Rosenblueth, M.; Martínez, L.; Silva, J.; Martínez-Romero, E., Klebsiella variicola, a novel species with clinical and plant-associated isolates. Systematic and applied microbiology 2004, 27, (1), 27-35.

[16] Engelhard, M.; Hurek, T.; Reinhold‐Hurek, B., Preferential occurrence of diazotrophic endophytes, Azoarcus spp., in wild rice species and land races of Oryza sativa in comparison with modern races. Environmental Microbiology 2000, 2, (2), 131-141.

[17] Reiter, B.; Bürgmann, H.; Burg, K.; Sessitsch, A., Endophytic *nif*H gene diversity in African sweet potato. Canadian journal of microbiology 2003, 49, (9), 549-555.

[18] Pırlak, L.; Köse, M., Effects of plant growth promoting rhizobacteria on yield and some fruit properties of strawberry. Journal of plant nutrition 2009, 32, (7), 1173-1184.

[19] deMelo, F. N.; S, I., Isolation and characterization of endophytic bacteria of coffee plants and their potential in caffeine degradation. Environmental toxicology 2006, 10, 293.

[20]Wheeler, C.; Miller, I., Current and potential uses of actinorhizal plants in Europe. The biology of Frankia and actinorhizal plants 1990, 365, 389.

[21]Belimov, A.; Dodd, I.; Safronova, V.; Hontzeas, N.; Davies, W., Pseudomonas brassicacearum strain Am3 containing 1-aminocyclopropane-1-carboxylate deaminase can show both pathogenic and growth-promoting properties in its interaction with tomato. Journal of Experimental Botany 2007, 58, (6), 1485-1495.

[22]Kennedy, I. R.; Tchan, Y.-T., Biological nitrogen fixation in non-leguminous field crops: Recent advances. Plant and Soil 1992, 141, (1), 93-118.

[23] Erturk, Y.; Ercisli, S.; Haznedar, A.; Cakmakci, R., Effects of plant growth promoting rhizobacteria (PGPR) on rooting and root growth of kiwifruit (Actinidia deliciosa) stem cuttings. Biological Research 2010, 43, (1), 91-98.

[24]Chen, W.-M.; James, E. K.; Prescott, A. R.; Kierans, M.; Sprent, J. I., Nodulation of Mimosa spp. by the β-proteobacterium Ralstonia taiwanensis. Molecular Plant-Microbe Interactions 2003, 16, (12), 1051-1061.

[25] Kennedy, I. R.; Choudhury, A.; Kecskés, M. L., Non-symbiotic bacterial diazotrophs in crop-farming systems: can their potential for plant growth promotion be better exploited? Soil Biology and Biochemistry 2004, 36, (8), 1229-1244.

[26]Belimov, A.; Hontzeas, N.; Safronova, V.; Demchinskaya, S.; Piluzza, G.; Bullitta, S.; Glick, B., Cadmium-tolerant plant growth-promoting bacteria associated with the roots of Indian mustard (Brassica juncea L. Czern.). Soil Biology and Biochemistry 2005, 37, (2), 241-250.

[27] Reeve, E. C., Encyclopedia of genetics[M]. Routledge, 2014.

[28]Hayat, R.; Ali, S.; Amara, U.; Khalid, R.; Ahmed, I., Soil beneficial bacteria and their role in plant growth promotion: a review. Annals of microbiology 2010, 60, (4), 579-598.

[29]Tilak, K.; Ranganayaki, N.; Pal, K.; De, R.; Saxena, A.; Nautiyal, C. S.; Mittal, S.; Tripathi, A.; Johri, B., Diversity of plant growth and soil health supporting bacteria. Current science 2005, 136-150.

[30] Rodrı́guez, H.; Fraga, R., Phosphate solubilizing bacteria and their role in plant growth promotion. Biotechnology advances 1999, 17, (4-5), 319-339.

[31]Chabot, R.; Antoun, H.; Kloepper, J. W.; Beauchamp, C. J., Root colonization of maize and lettuce by bioluminescent Rhizobium leguminosarum biovar phaseoli. Applied and Environmental Microbiology 1996, 62, (8), 2767-2772.

[32]Siddiqui, Z. A., PGPR: prospective biocontrol agents of plant pathogens. PGPR: biocontrol and biofertilization 2005, 111-142
